# Cell-type-specific consequences of mosaic structural variants in hematopoietic stem and progenitor cells

**DOI:** 10.1038/s41588-024-01754-2

**Published:** 2024-05-28

**Authors:** Karen Grimes, Hyobin Jeong, Amanda Amoah, Nuo Xu, Julian Niemann, Benjamin Raeder, Patrick Hasenfeld, Catherine Stober, Tobias Rausch, Eva Benito, Johann-Christoph Jann, Daniel Nowak, Ramiz Emini, Markus Hoenicka, Andreas Liebold, Anthony Ho, Shimin Shuai, Hartmut Geiger, Ashley D. Sanders, Jan O. Korbel

**Affiliations:** 1 Genome Biology Unit, European Molecular Biology Laboratory, Heidelberg, Germany; 2 Department of Systems Biology, College of Life Science and Biotechnology, Yonsei University, Seoul, Republic of Korea; 3 Institute of Molecular Medicine, Ulm University, Ulm, Germany; 4 Department of Human Cell Biology and Genetics, School of Medicine, Southern University of Science and Technology, Shenzhen, China; 5 Molecular Medicine Partnership Unit (MMPU), European Molecular Biology Laboratory, University of Heidelberg, Heidelberg, Germany; 6 Bridging Research Division on Mechanisms of Genomic Variation and Data Science, German Cancer Research Center (DKFZ), Heidelberg, Germany; 7 Department of Hematology and Oncology, Medical Faculty Mannheim of the Heidelberg University, Mannheim, Germany; 8 Department of Cardiothoracic and Vascular Surgery, Ulm University Hospital, Ulm, Germany; 9 Department of Medicine V, Hematology, Oncology and Rheumatology, University of Heidelberg, Heidelberg, Germany; 10 Berlin Institute for Medical Systems Biology, Max Delbrück Center for Molecular Medicine in the Helmholtz Association (MDC), Berlin, Germany; 11 Berlin Institute of Health (BIH) at Charité–Universitätsmedizin Berlin, Berlin, Germany; 12 Charité–Universitätsmedizin Berlin, Berlin, Germany

**Keywords:** Genomics, Ageing, Genomic analysis

## Abstract

The functional impact and cellular context of mosaic structural variants (mSVs) in normal tissues is understudied. Utilizing Strand-seq, we sequenced 1,133 single-cell genomes from 19 human donors of increasing age, and discovered the heterogeneous mSV landscapes of hematopoietic stem and progenitor cells. While mSVs are continuously acquired throughout life, expanded subclones in our cohort are confined to individuals >60. Cells already harboring mSVs are more likely to acquire additional somatic structural variants, including megabase-scale segmental aneuploidies. Capitalizing on comprehensive single-cell micrococcal nuclease digestion with sequencing reference data, we conducted high-resolution cell-typing for eight hematopoietic stem and progenitor cells. Clonally expanded mSVs disrupt normal cellular function by dysregulating diverse cellular pathways, and enriching for myeloid progenitors. Our findings underscore the contribution of mSVs to the cellular and molecular phenotypes associated with the aging hematopoietic system, and establish a foundation for deciphering the molecular links between mSVs, aging and disease susceptibility in normal tissues.

## Abstract (web summary)

This study uses Strand-seq to explore the landscape of mosaic structural variants (mSVs) in human hematopoietic stem and progenitor cells from people of different ages. The analysis highlights patterns of enrichment for mSVs in specific cell types, with associated phenotypes, and suggests that clonal expansions due to mSVs are generally restricted to older individuals.

## Main

Somatic subclonal (mosaic) mutations are present in nearly all tissues and accumulate with age^1–6^, yet their role in human health and disease is underexplored. Somatic structural variants, which comprise copy-number alterations (CNAs) and copy-neutral rearrangement classes, are the most common class of driver mutation in cancer^7,8^. Previous studies have associated mosaic CNAs in aged donors with unusual blood cell counts and susceptibility to age-associated diseases^2,9–12^, which underscores the potential for mSVs to alter molecular phenotypes in healthy individuals upon aging. However, the molecular processes behind these associations, which are anticipated to vary by cell type, are poorly understood.

Detecting mSVs poses an important technical challenge^7,11^, with bulk whole-genome sequencing (WGS) typically unable to differentiate cell types and identify mSVs present with a low variant allele frequency (VAF). Additionally, WGS of single-cell-derived clones is limited to mSVs that can be cultured long-term, potentially biasing against mSVs exhibiting large segmental aneuploidies^7,13,14^. Single-cell sequencing offers a solution in theory, yet most methods are suited only for detecting large CNAs, yielding an incomplete understanding of mSVs^15^.

Here we utilize Strand-seq, a haplotype-resolved single-cell sequencing technique^14,16,17^, to investigate the functional impact of mSVs. We focus on the blood compartment, where mosaic CNAs have been documented in aged donors^2,11,18,19^. Strand-seq allows resolving of diverse mSV classes, including de novo structural rearrangements, by analyzing their unique ‘diagnostic footprints’ utilizing the scTRIP framework^14^. Additionally, Strand-seq simultaneously yields nucleosome occupancy profiles from each single cell, generated via micrococcal nuclease (MNase) digestion^16^, which can be used to analyze the functional consequences of structual variants with the scNOVA framework^20^. In 1 of every 43 hematopoietic stem and progenitor cells (HSPCs), we detect de novo mSVs, which emerge regardless of age. We resolve the cell-type identity of mSV-bearing cells, revealing they are commonly enriched in myeloid progenitors and exhibit aberrant pathway activity previously associated with aging.

## Results

### Single-cell-resolved mSV landscapes in HSPCs

**Fig. 1:**
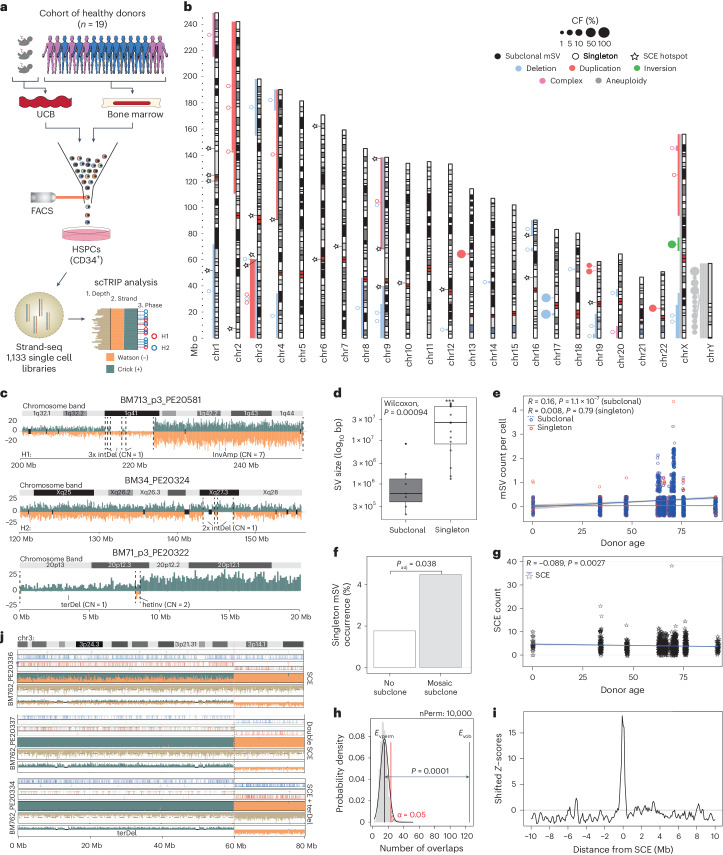
HSPCs acquire a wide diversity of mSVs with age, without increased chromosomal instability. **a**, Cohort and experimental workflow used. For visualization purposes, here and below, strand- and haplotype-specific DNA reads are colored as follows: Watson (−) reads, orange; Crick (+) reads, blue; SNPs phased to haplotype 1 (H1), red circles; SNPs phased to haplotype 2 (H2), blue circles. **b**, Genome-wide karyogram of mSVs identified. Bars indicate the size of identified mSVs, color indicates the class and the relative size of the bubble linked to the middle of each mSV depicts its cell fraction (CF). Filled circles denote subclonal mSVs, while unfilled ones are singleton mSVs. Stars indicate bins significantly enriched for SCEs. **c**, Examples of singleton complex mSVs identified in the cohort. Copy-number estimates in affected regions are shown next to the respective segments. Black dotted lines represent mSV breakpoints. DNA reads are colored as described in panel **a**. IntDel, interstitial deletion; InvAmp, inverted amplification; terDel, terminal deletion; hetInv, heterozygous inversion. **d**, Singleton mSVs (*n* = 67 examined over 10 independent donors) are significantly larger, when comparing mean total affected base pairs, than subclonal mSVs (two-sided Wilcoxon rank-sum test; *n* = 10 examined over 6 independent donors; boxplots were defined by: minima, 25th percentile − 1.5× interquartile range (IQR); maxima, 75th percentile + 1.5× IQR; center, median; and bounds of box, 25th and 75th percentiles). **e**,**g**, Jitter plots depicting trends in the number of subclonal and singleton mSVs (**e**), and SCEs (**g**), across age (*R*, correlation coefficient calculated from the number of mSVs/SCEs given the donor age; *P* value is based on the two-sided significance test for the Pearson correlation coefficient, testing the hypothesis that it is 0.). **f**, Barplot of the incidence of singleton mSVs (*y* axis) in cells with or without subclonal mosaicism. *P*_adj_ computed using two-sided Fisher’s exact tests. **h**, Results of the one-sided permutation test shuffling singleton mSV breakpoints (100-kb confidence interval) and SCE hotspots (200-kb bin) genome-wide for 10,000 permutations. The *P* value shows the significance of the difference between the permuted (black line) and actual (green) number of overlaps. **i**, Local *Z*-score of enrichment of overlaps between singleton mSV breakpoints and SCE hotspots. mSV breakpoints are shifted in windows of 100 kb to 10 Mb ±the bin in which an SCE hotspot is located, and the enrichment *Z*-score plotted each time. Additional permutations are plotted in Extended Data Fig. 1. **j**, Strand-seq data showing recurrent SCE and mSV co-occurrence at the SCE hotspot and *FRA3B* CFS in donor BM762. Haplotype-specific DNA reads and SNPs phased to H1 and H2 are colored as described in panel **a**. CN, copy number; *E*_vob_, observed overlaps; *E*_vperm_, expected overlaps; nPerm, number of permutations.

To study mSV formation in HSPCs with cell-type-specific resolution, we analyzed cells from 19 healthy donors—ranging from newborn to 92 years of age—composed of *n* = 3 umbilical cord blood (UCB) and *n* = 16 bone marrow samples (Fig. 1a). We isolated viable CD34^+^ HSPCs (Supplementary Fig. 1) and cultured them for one cell division to enable Strand-seq (Methods). We obtained 1,133 high-quality single-cell libraries, with a mean of 432,282 uniquely mapped fragments per cell (Supplementary Fig. 2 and Supplementary Table 1). We used scTRIP^14^ to discover mSVs and whole chromosome aneuploidies (herein, collectively called ‘mosaicisms’), both in single cells and in subclones. Altogether, we identify 51 independently arisen mosaicisms, occurring in 16 of 19 (84%) donors (mean per donor = 2.7; range 0–8), including: 22 deletions, 12 duplications, 3 complex mSVs involving three or more breakpoints, 1 balanced inversion and 13 chromosomal losses (Fig. 1b and Supplementary Table 2). These mosaicisms affect 17 of 24 chromosomes and exhibit no chromosomal enrichment except for the Y chromosome, which was independently lost once or multiple times (leading to mosaic loss of Y (LOY)) in 8 of 12 (67%) male donors.

Investigating the subclonal composition of each mosaicism (Supplementary Table 2), we find 32 that are detected in only 1 cell (‘singleton mosaicism’), while the remaining mSVs constitute subclones with a cell fraction (CF) of 1.6–56.1% (‘subclonal mosaicism’). While subclones with sex chromosome losses (*n* = 12 LOY; *n* = 1 loss of X) reach CFs up to 46.4%, we do not observe whole autosomal aneuploidies. Focusing our further investigation on the 38 autosomal mSVs, we find notable differences between singleton and subclonal mosaicisms. First, 21 of 31 singleton mSVs (68%) exhibit terminal gains or losses, whereas all seven subclonal mSVs comprise interstitial alterations. Second, all complex mSVs are singletons. These include a breakage fusion bridge cycle-mediated^14^ mSV on chromosome 20p, and a terminal amplification of 1q (Fig. 1c). Third, singleton mSVs are nearly 18 times larger on average than subclonal mSVs (36.9 versus 2.1 megabase pairs (Mb), respectively; *P* = 0.0009, Wilcoxon rank-sum test; Fig. 1d). These data indicate that singleton mSVs, detected in 1 of every 43 HSPCs, bear the characteristics of de novo rearrangements (Supplementary Notes), suggesting that not all mSVs have the same potential to form appreciable subclones.

Analyzing these data with respect to donor age shows subclonal mSV expansions (Pearson’s correlation; *R* = 0.16; *P* = 1.1 × 10^−7^) and sex chromosome losses (*R* = 0.087; *P* = 0.0034) are associated with increased age (Fig. 1e), consistent with previous studies of mosaic CNAs^2,11,18,19^. Conversely, singleton mSVs are uncorrelated with age (*R* = 0.008; *P* = 0.79; Fig. 1e), suggesting continuous acquisition throughout life. Instead, we observe elevated numbers of de novo mSVs in cells already containing a subclonal mosaicism versus unmutated cells (Fisher’s exact test; 4.76% versus 1.96%; *P* = 0.038; Fig. 1f), suggesting that mSV-harboring cells may be ‘predisposed’ to accumulate further rearrangements.

### Hotspots of mSV formation

**Extended Data Fig. 1:**
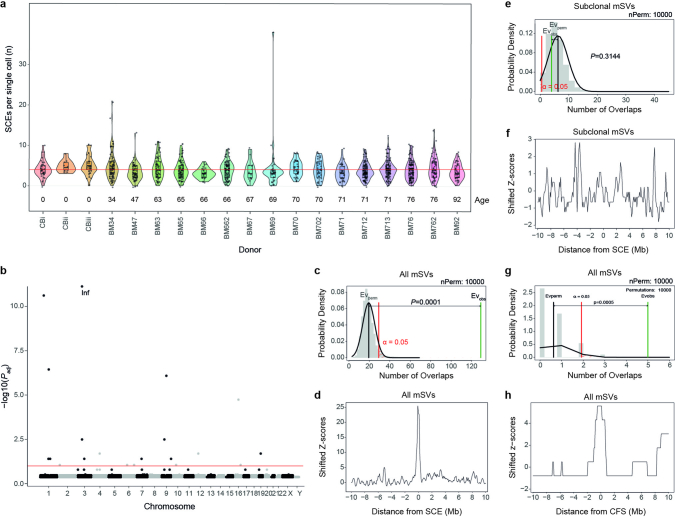
SCEs occur steadily over life but arise non-randomly across the genome. Violin plot showing the number of SCEs per single cell per donor, in order of increasing donor age. The age of each donor is noted below the violin in each case. The median SCE count (n = 4 SCEs) is indicated by a red line. Boxplots were defined by minima = 25th percentile - 1.5X interquartile range (IQR), maxima = 75th percentile + 1.5X IQR, center = median, and bounds of box = 25th and 75th percentile. **b**) Manhattan plot showing the distribution of SCEs genome-wide (hg38, 500 kb bins). Red line denotes the significance cutoff used (10% FDR). The significance of SCE counts per bin were calculated by fitting the permuted data of the number of overlaps per bin genome-wide (quantified using bedtools intersect) to a negative binomial distribution using the fitdist() function from the fitdistrplus package^83^ (evaluation of distribution of empirical vs theoretical data shown in Supplementary Fig. 4), and then computing the p-value of the actual data using the fitted negative binomial distribution as a null distribution. The resulting size and mu (size = 1.4013518 (standard error = 0.08457053), mu = 0.6714258 (standard error = 0.01213493)) were used to transform SCE counts per bin into p-values, followed by Benjamini-Hochberg correction^85^ to control the FDR. **c**, **e**) Permutation summary plots for 10,000 permutations of breakpoint regions from **c**) all mSVs and **c**) subclonal mSVs vs. all SCE locations. **d, f**) Local Z-score plots showing enrichment Z-scores within a 20 Mb window, in 2 Mb bins, for **d**) all mSVs and **f**) subclonal mSVs. **g**) Permutation summary plots for 10,000 permutations of breakpoint regions from all mSVs vs. previously annotated CFSs^86^. P-value is based on a one-sided permutation test. **h**) Local Z-score plots showing enrichment Z-scores within a 20 Mb window, in 2 Mb bins, for all mSVs vs previously annotated CFS regions.

Since DNA double-strand breaks (DSBs) can trigger structural rearrangements^7,21^, we examined the correlation between DSB acquisition and donor age. Strand-seq enables the detection of sister chromatid exchanges (SCEs) to allow systematic mapping of DSBs following homologous repair^16^. We identified 4,528 SCEs in our dataset (~4 SCEs per cell, consistent with previous reports^22^; Extended Data Fig. 1a). SCE abundance is inversely correlated with age (*R* = −0.089; *P* = 0.0027; Fig. 1g and Supplementary Fig. 3), with on average 4.6 SCEs per cell in individuals <60, compared with 3.9 SCEs per cell in donors >60 (Extended Data Fig. 1a). With HSPCs exhibiting largely stable acquisition of mSVs and SCEs regardless of age, these data suggest mSV formation occurs consistently throughout life.

Since structural rearrangements can be influenced by local sequence context^7^, we analyzed the genomic locations of SCEs and mSVs. The skewed distribution of SCEs along chromosomes is even more pronounced than that of mSVs (Fig. 1b and Supplementary Fig. 4): 6.67% (302 of 4,528) cluster into 20 SCE ‘hotspots’ (Methods, Extended Data Fig. 1b and Supplementary Table 3), of which five (25%) coincide with common fragile sites^23^ (CFSs) (Supplementary Table 4). Notably, SCEs overlap significantly with mSV breakpoints, with 3% (133 of 4,528) of all SCEs intersecting an mSV breakpoint (*P* < 0.0001, derived from 10,000 permutations; Fig. 1h,i, Extended Data Fig. 1c–f and Supplementary Table 3). While CFSs are enriched for both SCEs (*P* < 0.0002) and mSV breakpoints (Extended Data Fig. 1g,h), we identify additional SCE hotspots with similar enrichments not previously identified as CFSs (Fig. 1b,j, Supplementary Fig. 5 and Supplementary Tables 2–4). These loci may therefore represent mSV hotspots in HSPCs.

### High-precision cell-typing using nucleosome occupancy profiles

**Fig. 2:**
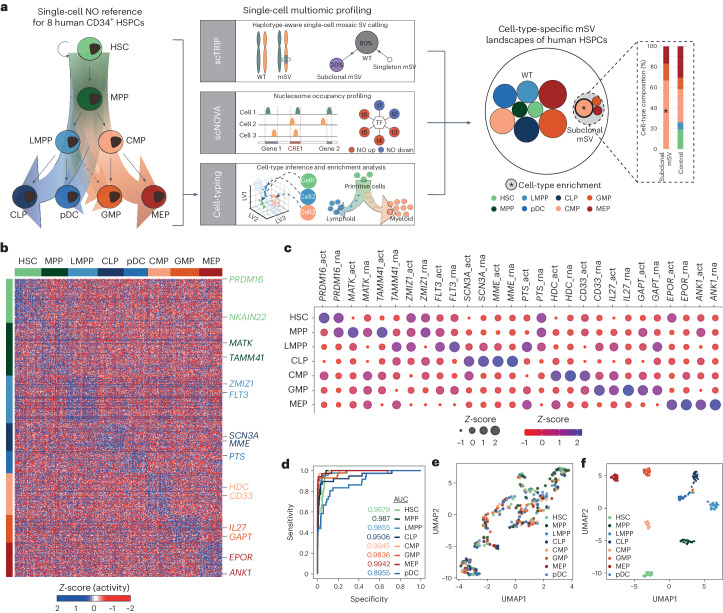
scMNase-seq atlases for eight distinct HPSCs enable cell-type-aware single-cell multiomic profiling. **a**, Single-cell multiomic analysis workflow used to investigate mSVs in HSPCs with Strand-seq, which involves single-cell mSV discovery (scTRIP^14^), single-cell nucleosome occupancy (NO) analysis to infer mSV functional effects (scNOVA^20^) and cell-typing. GMP, granulocyte–macrophage progenitor; pDC, plasmacytoid dendritic cell. **b**, Construction of bone marrow and UCB-specific NO reference datasets to allow for cell-typing, based on subjecting HSPC cell types to index sorting, and scMNase-seq. Heatmap of single-cell NO of gene bodies of 305 single bone marrow HSPCs (UCB-based reference shown in Extended Data Fig. 2). The 819 signature genes depicted (rows) allow for discrimination between eight cell types (columns). Cells are grouped and color-coded by immunophenotype, determined by FACS. Example marker genes for each cell type are shown to the right of the heatmap, color-coded by the cell type. Differential NO of marker genes is represented by *Z*-scores. **c**, Comparison of inferred gene activity^20^ (act), based on inverse NO and publicly available gene expression (RNA sequencing) data^24^ for the representative classifier genes from the bone marrow scMNase-seq reference. Gene activity at gene bodies was inferred using the NO *Z*-score multiplied by (−1). Color and the dot sizes reflect the *Z*-score of inferred gene activity and RNA expression, respectively. **d**, Receiver operating characteristic (ROC) curve showing leave-one-out cross-validation of the bone marrow cell-type classifier’s performance using single-cell NO patterns. **e**, Unsupervised UMAP dimensionality reduction of the bone marrow HSPC scMNase-seq data. **f**, Supervised UMAP dimensionality reduction of the data in **e**, using the bone marrow cell-type classifier. AUC, area under the curve.

To investigate the cell-type-specific impact mSVs exert on HSPCs, we utilized a two-pronged approach by coupling single-cell mSV analysis with nucleosome occupancy-based functional profiling^20^. First, to develop nucleosome occupancy-based cell-type classifiers^20^, we constructed single-cell nucleosome occupancy reference profiles for HSPCs derived from both UCB and bone marrow, covering eight distinct cell types: hematopoietic stem cells (HSCs), multipotent progenitors (MPPs), lymphoid-primed multipotent progenitors (LMPPs), common lymphoid progenitors (CLPs), plasmacytoid dendritic cells, common myeloid progenitors (CMPs), granulocyte–macrophage progenitors and megakaryocyte–erythroid progenitors (MEPs) (Fig. 2a and Supplementary Fig. 6). Using well-defined immunophenotypes (Supplementary Table 5 and Supplementary Fig. 6) we index-sorted HPSCs, and devised a preamplification-free single-cell MNase sequencing (scMNase-seq) protocol (Methods) to characterize the single-cell nucleosome occupancy profile for each cell type.

**Extended Data Fig. 2:**
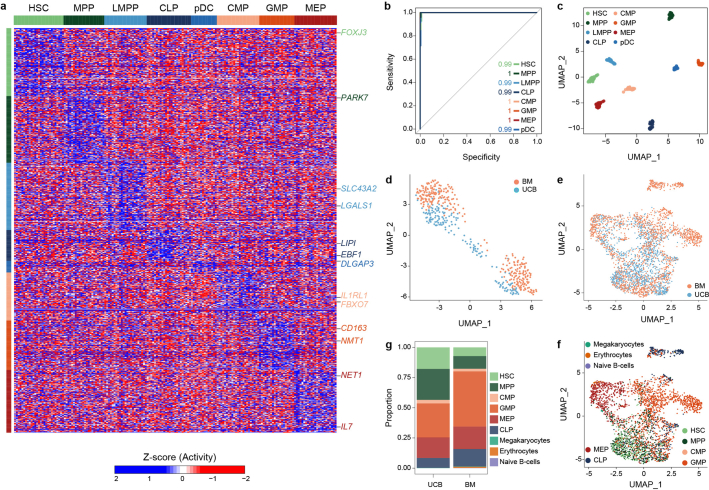
Performance evaluation of the UCB-derived scMNase-seq-based cell-type classifier. **a**) ROC curve showing leave-one-out cross-validation of the cell-type classifier’s performance using single cell NO patterns. **b**) UMAP projection of latent variables from the UCB HSPC cell type classifier. **c**) Heatmap of single cell NO of gene bodies of 175 single UCB HSPCs, generated using scMNase-seq. The 899 signature genes depicted (rows) allow for discrimination between 8 UUCB HSPC cell types (columns). Cells are grouped and color-coded by immunophenotyped cell-type identity, determined by FACS (Supplementary Fig. 6). Differential NO of marker genes is represented by Z-scores. **d**) UMAP projection of scMNase-seq latent variables, coloured by tissue-of-origin. BM, bone marrow. **e**) UMAP of single-cell transcriptome data of HSPCs from UCB and adult bone marrow, obtained from^27^. **f**) Cell-types in the scRNA-seq were annotated using the singleR package^72^ based on the blueprint ref. ^87^. **g**) Cell-type composition of scRNA-seq from UCB and BM shows that MPPs are highly enriched in UCB compared to BM HSPCs. MPPs, CLPs, and pDCs exhibit a lower prevalence than other cell-types, likely reflecting their natural scarcity in HSPCs^24^ and known challenges with sustaining these cells *in vitro*^88^.

We obtained 480 high-quality scMNase-seq libraries (Supplementary Table 6): 305 from bone marrow-derived HSPCs (1 donor) and 175 from UCB-derived HSPCs (5 donors) (Supplementary Table 1). Using scNOVA, we identify 899 and 819 genes exhibiting cell-type-specific nucleosome occupancy in the UCB- and bone marrow-derived datasets, respectively (Fig. 2b and Extended Data Fig. 2a). The cell-type-specific gene activities inferred from nucleosome occupancy^20^ are broadly consistent with published transcriptomic datasets^24^ (Fig. 2c). For example, from the bone marrow-derived nucleosome occupancy dataset, we infer increased activity of the canonical marker *MME* (CD10) only in CLPs^25^, while *HDC* (involved in myeloid-lineage priming^26^) exhibits increased activity in CMPs. We also observe differential nucleosome occupancy at genes not previously reported as HSPC markers, such as *SH2D4B* and *FAT3* (Supplementary Table 7a,b).

Harnessing these gene sets, we utilized nucleosome occupancy measurements as features for developing supervised cell-type classification models using partial linear square discriminant analysis (PLS-DA) (Fig. 2d–f, Extended Data Fig. 2, Supplementary Table 7a,b and Methods). These classifiers provide excellent accuracy, with an average area under the curve of 0.97 for bone marrow and 1.00 for UCB, as estimated by leave-one-out cross-validation (Fig. 2d and Extended Data Fig. 2). Uniform manifold approximation and projection (UMAP) of the latent variables corroborate the discriminatory power of these classifiers compared with unsupervised classification (Fig. 2e,f and Extended Data Fig. 2).

### Subclonal mSVs commonly exhibit a lineage bias

**Fig. 3:**
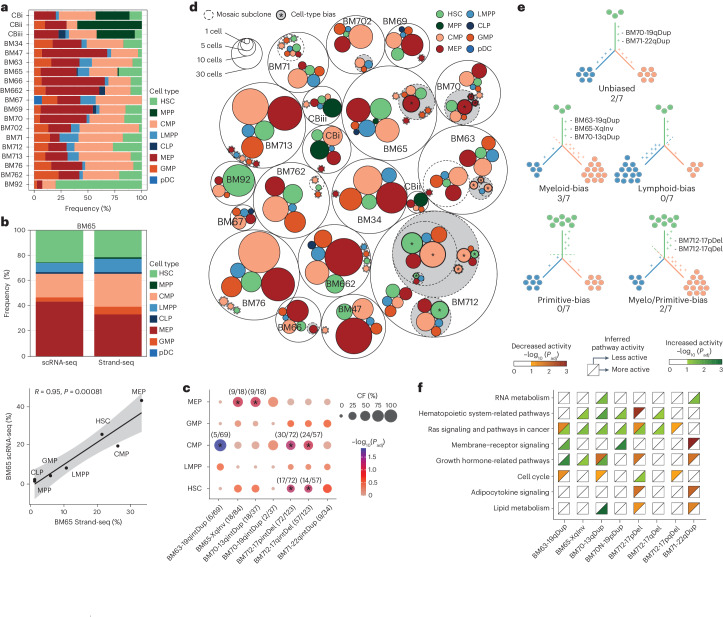
mSVs in HSPCs frequently exhibit a cell-type bias. **a**, Inferred cell-type composition (based on Strand-seq) per donor (ordered by age). **b**, Upper, stacked bar graph depicting the HSPC cell-type composition in BM65, estimated through SingleR cell-type annotation^72^ of scRNA-seq, utilizing previously published immune cell-type annotations as a reference profile^51,57^, compared with cell-typing of Strand-seq data. Lower, cell-type compositions are highly correlated between Strand-seq (*x* axis) and scRNA-seq (*y* axis) in BM65. The error band indicates the confidence interval controlling the 95% confidence region. (*R*, correlation coefficient calculated from the *x* and *y* axes; *P* value is based on the two-sided significance test for the Pearson correlation coefficient, testing the hypothesis that it is 0.) **c**, Dotplot of results of the cell-type enrichment analysis for each mSV identified, showing the CF, enrichment and significance of each cell type per mSV subclone versus an idealized control. The number in brackets indicates the number of single cells of a given cell type, in a given subclone, used to calculate enrichment. Data here show enrichment for single genotypes; for combined enrichments see Supplementary Fig. 10. **d**, Circle-packing plot summarizing the mSVs and inferred cell-type composition of each subclone for each of the 19 donors. Transparent circles with a solid outline represent distinct samples. Transparent inner circles with dashed outlines represent mosaicism-bearing subclones within a sample, while colored circles denote the cell types contributing to the subclone. Each circle is proportional to the total number of single cells composing that cell type/subclone. A gray background identifies mosaicism-bearing subclones showing a significant (FDR 10%) cell-type enrichment with respect to the control group of karyotypically normal cells. **e**, Summary of lineage biases observed across all subclonal mSVs (that is, excluding LOY/loss of X) across the cohort. **f**, Enrichment analysis of pathways grouped by Jaccard similarity, for subclonal mSVs across the cohort. Only groups of pathways enriched in two or more mSVs are shown. For all individual pathways, see Supplementary Fig. 12. For all groups of pathways and details on Jaccard similarity-based grouping, see Supplementary Fig. 35.

Having constructed nucleosome occupancy references for HSPCs, we next performed cell-typing of each Strand-seq library (Fig. 3a and Supplementary Table 8). Tissue-level cell abundances detected based on nucleosome occupancy show high consistency with previous studies^24,27–29^, including an expanded HSC frequency in older bone marrow donors (from 8.1% to 80%; false discovery rate (FDR)-adjusted *P* (*P*_adj_) = 0.013; mixed linear model analysis), and a greater abundance of MPPs in UCB versus bone marrow^24,27^ (37% versus 0.1%; *P*_adj_ = 2.45 × 10^−33^; Fisher’s exact test; Extended Data Fig. 2). Furthermore, the cell-type compositions seen in Strand-seq closely resemble estimates from orthogonal single-cell RNA sequencing (scRNA-seq) data generated from two donors (BM65, BM712), independently verifying our nucleosome occupancy-based classifiers (Fig. 3b and Supplementary Fig. 8).

We next explored the cellular context of mSVs. Of the 19 subclonal mosaicisms found, 8 (42%) show significant cell-type enrichments (FDR 10%; Fig. 3c,d and Supplementary Figs. 9 and 10); and, when considering only subclonal mSVs (that is, removing sex chromosome losses), 5 of 7 (71%) show significant biases. Here, we find predominantly myeloid skewing, with 5 of 5 (100%) of the cell-biased subclonal mSVs enriched in either myeloid or myelo-primitive cell types (Fig. 3e). These lineage-biased events include: a 10-Mb inversion on chromosome Xq12-Xq21.1 enriched in MEPs (BM65); a 1-Mb duplication at 13q enriched in MEPs (BM70); a 300-kilobase (kb) duplication at 19q enriched in CMPs (BM63); and two sequentially arisen deletions at 17p (1.2 Mb) and 17q (500 kb) enriched in both CMPs and HSCs (BM712).

By comparison, sex chromosome losses exhibit more variability, with cell-type enrichments seen in only 3 of 12 (25%; all LOYs) and each of these exhibiting bias for a different cell type: MEP, LMPP and HSC, respectively (Supplementary Figs. 10 and 11). This suggests that the functional impact of LOY is less pronounced or more context-specific^30^. Furthermore, singleton mSVs do not show cell-type enrichment (Supplementary Fig. 10), suggesting that lineage biases seen in subclonal mSVs are due to their impact on cellular function, rather than biased acquisition in a specific cell type.

Remarkably, despite the diverse genomic loci affected by subclonal mSVs, there is a notable convergence on certain molecular phenotypes. Specifically, the Ras and JAK/STAT signaling pathways, as well as lipid metabolism—previously associated with clonal hematopoiesis (CH) and leukemia^31,32^—are recurrently altered (Fig. 3d–f, Supplementary Figs. 12 and 13 and Supplementary Tables 9, 10 and 17). These data link mSVs to common changes in aging-related pathways.

### Cell-type-specific impact of an inversion

**Fig. 4:**
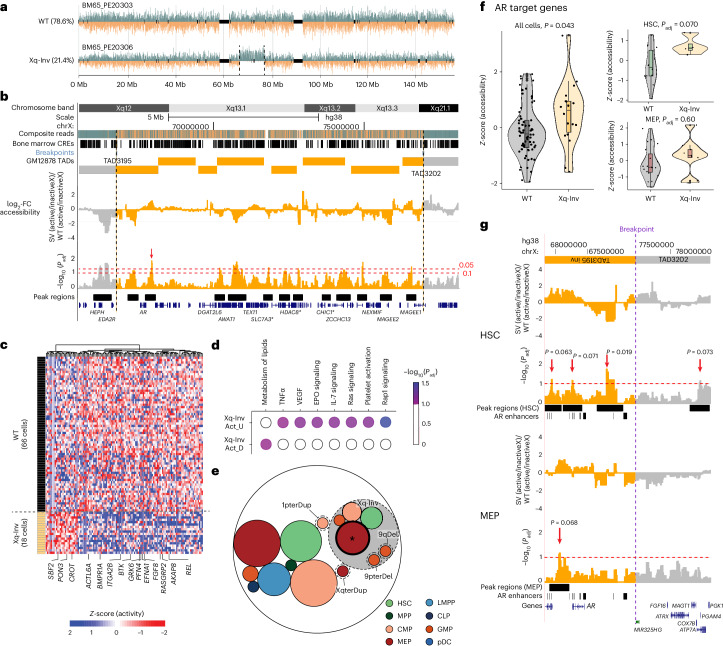
Mosaic inversion contributes to MEP-biased cell fate and subclonal expansion of HSPCs through *cis* and *trans* effects. **a**, Strand-seq data of X chromosomal homologs from BM65 depicting the unaffected haplotype 2 (also denoted ‘WT’; top) and the Xq-Inv (somatic mosaic inversion on chromosome Xq) on haplotype 1 (bottom) in single cells. For visualization purposes, here and below, strand- and haplotype-specific DNA reads are colored as follows: Watson (−) reads, orange; Crick (+) reads, blue. **b**, Genome browser track showing the confidence interval of inversion breakpoints and annotated TAD boundaries^73^ around them. Below, NO differences at CREs between Xq-Inv and WT cells are shown as log_2_-fold changes (permutation-adjusted *P* values computed using a sliding window approach^20^). The most significant signal out of 13 peaks representing patterns of haplotype-specific NO is a region with inferred increased chromatin accessibility, which overlaps with annotated *AR* enhancers^74^ residing 386 kb apart from the *AR* gene. Three annotated *AR* enhancers intersecting with the most significant peak are highlighted in red. The black vertical dotted lines indicate the breakpoint positions of mSVs, and the red horizontal dotted lines show the significance level of haplotype-specific NO (FDR 5%, and 10%). **c**, Heatmap of differential nucleosome occupancy (diffNO) genes identified in Xq-Inv cells compared with WT cells, generated after regressing out the contribution of individual cell types. The *y* axis represents single cells analyzed, and diffNO genes are plotted on the *x* axis. Changes in inferred gene activity are colored from red (increased gene activity) to blue (decreased gene activity). **d**, Pathways over-represented by the genes with diffNO (FDR 10% based on the hypergeometric test; Act_U, activity up; Act_D, activity down). **e**, Circle-packing plot depicting cell-type-resolved mSVs (terDup, terminal duplication; terDel, terminal deletion). Dotted lines denote mSVs; gray-colored background denotes measured cell-type enrichment. **f**, Violin plot of NO of known AR target genes, which exhibit an AR-binding motif in their promoter based on MsigDB^75^, in Xq-Inv (*n* = 18 cells) and WT cells (*n* = 66 cells), all cell types (left), HSCs only (*n* = 18 cells, upper-right) and MEPs (*n* = 28 cells, lower-right). Boxplots were defined by minima, 25th percentile − 1.5× IQR; maxima, 75th percentile + 1.5× IQR; center, median; and bounds of box, 25th and 75th percentiles. *P* values are based on the two-sided likelihood ratio test followed by Benjamini–Hochberg multiple correction. The gray and yellow shading of violin plots show the genotype of cells (gray, WT; yellow, Xq-Inv). **g**, Cell-type-specific analysis of NO differences at CREs between the mSV subclone and WT cells. *P*_adj_ values of significant peak regions (FDR < 10%) are highlighted. A red arrow indicates the HSC-specific significant peak region containing two AR enhancers, in which we infer increased chromatin accessibility (these two enhancers are highlighted in red in Supplementary Fig. 18). The red dotted lines indicate the significance level of haplotype-specific NO (FDR 10%).

The molecular consequences of mosaic inversions are underexplored, since most studies are biased towards CNAs^7,11^. We therefore investigated the Xq12-Xq21.1 inversion (‘Xq-Inv’), seen in 22.6% (19 of 84) of cells from a 65-year-old female donor (BM65; Fig. 4a). Nucleosome occupancy analysis^20^ confirms the inversion lies on the active X-homolog (Supplementary Fig. 14), supporting its potential for mediating functional effects. We refined the inversion breakpoints^33^ (Methods) to chrX:66753519–76960327, with confidence intervals of ~10 kb and ~18 kb, respectively. While neither breakpoint directly overlaps a gene, the inversion is predicted to fuse two topologically associating domains (TADs) by disrupting their annotated boundaries (Fig. 4b), putatively altering the respective gene regulatory environments^34^.

To investigate the potential impact of the inversion, we interrogated haplotype-resolved nucleosome occupancy profiles at *cis*-regulatory elements (CREs) to infer chromatin accessibility for each homolog^20^. Using a haplotype-aware sliding window analysis (Methods), we normalized nucleosome occupancy between the active and inactive X, and compared Xq-Inv cells with unmutated cells from the same donor. We identify 13 peak regions with significantly altered nucleosome occupancy (10% FDR; Fig. 4b), with 4 (31%) located within one of the affected TADs. The strongest peak fell into an intergenic region and showed decreased nucleosome occupancy on the inverted haplotype, indicating increased chromatin accessibility^20^. This peak is located adjacent to the androgen receptor gene (*AR*). Closer analysis shows three annotated *AR* enhancers fall within this peak (Supplementary Table 11), all residing in the fused TAD (Fig. 4b and Supplementary Fig. 14). These data suggest *AR* as a potential target of gene dysregulation and contributor to subclonal expansion. Indeed, androgens are used to treat bone marrow failure syndromes by inducing HSPC proliferation, albeit with an incompletely understood mode of action^35^.

To study the downstream effects of the Xq-Inv, we performed a genome-wide search for differential gene activity^20^, comparing the nucleosome occupancy of gene bodies between Xq-Inv and unmutated cells (Methods). We find 123 genes displaying differential nucleosome occupancy (Fig. 4c and Supplementary Table 10)—all of which reside outside the inversion locus—suggesting strong *trans* effects of Xq-Inv. Gene set over-representation analysis reveals dysregulation of several AR-related pathways, including Ras signaling and erythropoietin signaling (10% FDR; Fig. 4d and Supplementary Table 12). Erythropoietin signaling, for example, contributes to an erythroid-bias of HSCs in association with elevated *AR* activity^36,37^. Finally, TF-target enrichment analysis^20^ reveals three TFs with differential activity in Xq-Inv cells: *EGR1*, *RUNX1* and *IKZF1*—all of which are linked to *AR* signaling (Supplementary Fig. 15). These data independently suggest *AR* activation as a result of Xq-Inv.

Notably, all three TFs have previously been reported to play critical roles in MEPs^38–40^, hinting that AR activation could be a key factor in the enrichment of MEPs within the Xq-Inv subclone (Fig. 4e). To explore this, we performed a cell-type-aware nucleosome occupancy analysis in the *AR* gene-body, revealing elevated *AR* activity from the rearranged homolog in HSCs, but not in MEPs (10% FDR; Supplementary Fig. 16). Likewise, upon testing *AR* target genes (Supplementary Table 13) we infer increased activity in HSCs, but not MEPs, with Xq-Inv (10% FDR; Fig. 4f and Supplementary Fig. 15), indicating HSC-specific *AR* overactivation in Xq-Inv cells. Consistent with this, Xq-Inv HSCs contain unique differential nucleosome occupancy peaks (10% FDR), including at two *AR* enhancers (Fig. 4g and Supplementary Fig. 17). These enhancers, which contain binding sites for EGR1, RUNX1 and IKZF1, are more accessible in HSCs, suggesting cell-type-specific enhancer activities (Supplementary Fig. 18). Finally, where these HSCs show regulatory changes consistent with elevated AR signaling (with 3 of 4 differential nucleosome occupancy genes representing annotated *AR* targets), Xq-Inv myeloid cells (CMPs and MEPs) show a more diffuse signal (with 23 of 105 and 12 of 55 differential nucleosome occupancy genes being *AR* targets, respectively) (Supplementary Table 9 and Supplementary Fig. 15). Among the MEP-specific genes, we infer high activity of *RIT1* (*P*_adj_ = 0.0057), a gene whose overexpression has been implicated in CH with MEP expansion^41^. Comparing the scRNA-seq data from BM65 with HSPCs from the Human Cell Atlas bone marrow cohort^42^ shows significant enrichment for AR activity in BM65 versus the Human Cell Atlas cohort in HSCs and MEPs, but not LMPPs (Supplementary Fig. 19). These findings are in line with androgen-mediating erythropoiesis through AR-dependent pathways^43^. They further imply HSC-specific AR overactivity, with a ‘priming’ role of Xq-Inv biasing cells towards megakaryocyte–erythroid lineages.

### Stepwise accumulation of mSVs in HSPCs

**Fig. 5:**
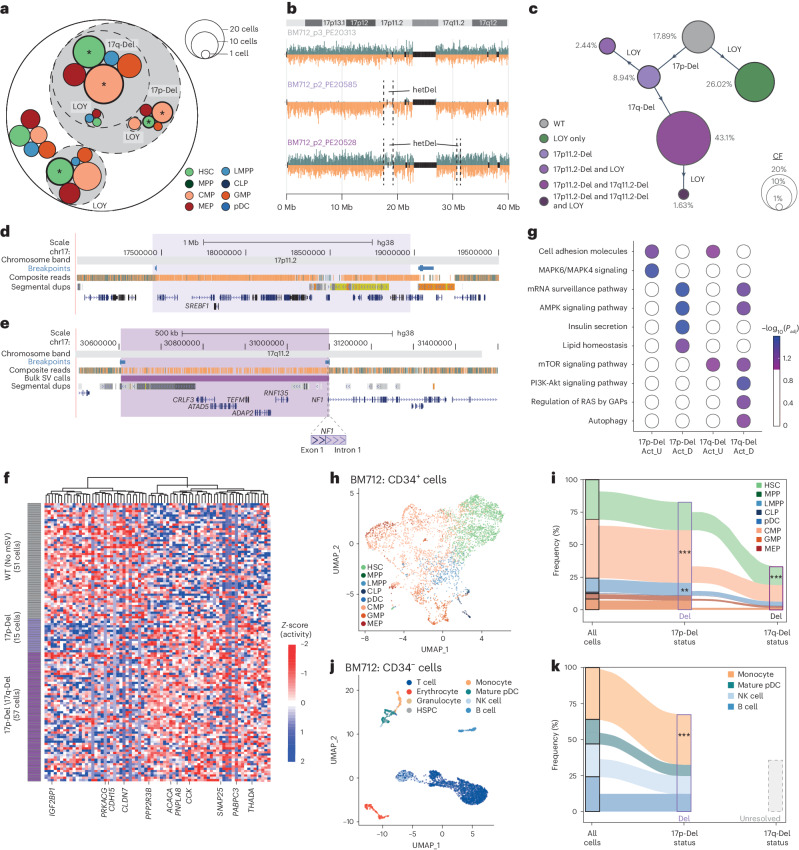
mSV accumulation in a single donor associates with clonal expansion. **a**, Circle-packing plot of mSVs found in BM712. **b**, Strand-seq karyograms of unmutated (WT; upper), 17p-Del (somatic mosaic heterozygous deletion on chromosome 17p) only (middle) and 17p-Del and 17q-Del (somatic mosaic heterozygous deletion on chromosome 17q) (bottom) somatic genotypes in single cells. **c**, Bubble hierarchy plot of mSVs identified in BM712. Bubbles are colored by somatic genotype, and scaled proportionally to each subclone’s frequency within the donor. CF is noted beside each bubble, and the distinguishing mosaicism acquired by each subclone indicated on the adjoining arm from the parent population. **d**,**e**, UCSC genome browser tracks for the 17p-Del (**d**) and 17q-Del (**e**) genomic segments. Tracks for both panels include composite read data and BreakpointR^33^-based breakpoint assignments, and highlight relevant genes. In **e**, the high-confidence deletion call from bulk WGS is also displayed (VAF inferred by Delly2 (ref. ^76^) is 28.5%). **f**, Heatmap of genes showing differential NO between WT, 17p-Del and 17pq-Del cells. **g**, Pathway over-representation analysis using ConsensusPathDB^77^ for the genes identified in the pairwise comparison of 17p-Del and 17pq-Del subclones with WT cells (FDR 10% based on the hypergeometric test). On the *x* axis, Act_U and Act_D depict increased and decreased activity, respectively. **h**, UMAP plot of scRNA-seq of CD34^+^ cells, with inferred cell type from reference data^51^ overlaid. **i**, Cell-type composition and enrichment analysis for 17p-Del and 17pq-Del subclones in scRNA-seq data of CD34^+^ cells. Asterisks indicate cell types with significant enrichment in a given subclone, based on Benjamini–Hochberg-adjusted Fisher’s exact test. **j**, UMAP plot of scRNA-seq of CD34^−^ cells, with cell type inferred from single-cell reference datasets^51,57^ overlaid. **k**, Cell-type composition and enrichment analysis for the 17p-Del subclone in scRNA-seq of CD34^−^ cells. ‘Unresolved’, the 17q-Del subclone could not be resolved in these scRNA-seq data owing to the low numbers of expressed genes covered. Significant cell-type enrichment with **P_adj_ < 0.001 or ***P_adj_ < 0.0001, respectively, based on two-sided Fisher’s exact test followed by Benjamini–Hochberg multiple testing correction. In CD34^+^ cells, CMPs and LMPPs are enriched in the 17p-Del subclone (*P*_adj_ = 1.99 × 10^−11^ and *P*_adj_ = 6.48 × 10^−3^, respectively) and HSCs are enriched in the 17q-Del subclone (*P*_adj_ = 2.07 × 10^−5^). In the case of CD34^−^ cells, monocytes are enriched in the 17p-Del subclone (*P*_adj_ = 9.6 × 10^−29^). dups, duplications; NK, natural killer.

While our data indicate that mSVs impact molecular phenotypes, how subclonal expansions are facilitated in cells harboring more than one co-existing mSV is unclear. We explored subclone dynamics in a 71-year-old male donor (BM712) exhibiting five distinct subclones, three of which demonstrate cell-type bias (FDR 10%; Fig. 5a). Of the 123 cells sequenced, 103 (84%) harbor at least one subclonal mosaicism, including two interstitial deletions and three LOYs (Fig. 5a,b). We tracked the subclonal evolution of BM712 using shared mSVs. One subclone (26% CF) shows LOY as the only mSV event and is enriched for HSCs. The four other subclones trace back to a ~1.2-Mb deletion at 17p11.2 (17p-Del), seen in 56% of cells, followed by the progressive acquisition of a ~500-kb deletion at 17q11.2 (17q-Del) and two independent LOYs (Fig. 5c–e). Bulk WGS of CD34^−^ cells verified the subclonal 17q-Del and 17p-Del events (Fig. 5e and Supplementary Fig. 20), and revealed both mSVs are carried into mature blood cells.

To explore the functional impact of the initiating mSV (17p-Del), we compared the gene-body nucleosome occupancy of 17p-Del cells with unmutated cells from BM712 using scNOVA, identifying 76 dysregulated genes (10% FDR; Fig. 5f). TF-target over-representation analysis^20^ shows enrichment for the targets of seven TFs, with the most significant being SREBF1 (*P*_adj_ = 0.0047) (Supplementary Fig. 21). This gene is hemizygously deleted by 17p-Del, while the other six TFs fall outside the deletion, suggesting a potential important role for *SREBF1* loss in the molecular phenotype of 17p-Del cells (Fig. 5d). Protein–protein interaction mapping of all seven dysregulated TFs using STRING^44^ (Supplementary Methods) reveals a significant protein–protein interaction network connecting all TFs (*P* = 3.57 × 10^−8^; Supplementary Fig. 21), highlighting their functional relationship (Supplementary Notes). Pathway enrichment analysis shows this network is enriched for MAPK signaling components (*P*_adj_ = 0.0028), previously linked to cell-cycle activation in aging HSCs^45^. Finally, gene set over-representation analysis of all 76 dysregulated genes supports MAPK activation (Fig. 5g), along with dysregulation of lipid homeostasis, a contributor to increased myelopoiesis^46^. Taken together, this suggests that 17p-Del triggers increased MAPK activity, potentially driving myeloid-biased clonal expansion through hemizygous *SREBF1* loss.

We next investigated the consequences of 17q-Del, seen in a subclone with 43.1% CF. This deletion disrupts the *NF1* tumor suppressor via hemizygous loss of protein-coding exon 1 (Fig. 5e and Supplementary Figs. 22 and 23). In addition to its well-understood roles in cancer^47^, *NF1* has been nominated as a CH driver by single nucleotide variant (SNV) analysis^48^ (Supplementary Notes), suggesting that the 17q-Del may fuel HSPC clonal expansion. Using scNOVA, we find 112 dysregulated genes in 17q-Del cells. Pathway over-representation analysis also shows altered metabolism and upregulated mTOR signaling in the subclone (Supplementary Fig. 24). Given the known critical role of *NF1* in mTOR signaling^49^, and the role of mTOR signaling in cell proliferation and HSPC differentiation^50^, these findings suggest that the 17q-Del induces mTOR dysregulation, potentially fostering subclonal expansion.

To further characterize these subclones, we generated 4,114 scRNA-seq libraries from CD34^+^ cells isolated from BM712 (Supplementary Fig. 25), and assigned HSPC cell types to the data using a transcriptome reference of human blood^51^ (Fig. 5h). To molecularly phenotype the deletion subclones, we capitalized on the fact that copy-number-imbalanced mSV classes can be utilized for targeted re-calling of CNAs in scRNA-seq data^20^ (Methods), allowing characterization of mSV-bearing cells across a widened dynamic expression range. Using this approach, we infer that 2,571 (63%) scRNA-seq cells bear the 17p-Del, 1,841 (45%) contain the 17q-Del and 995 (24%) exhibit LOY (Supplementary Table 14)—CFs similar to the Strand-seq analyses. Co-occurrence analyses of these mosaicisms corroborate the subclonal structure identified using Strand-seq (Supplementary Fig. 26). Finally, the scRNA-seq data also verify the inferred lineage biases, with 17p-Del cells enriched for CMPs and LMPPs (*P*_adj_ = 2.0 × 10^−11^, *P*_adj_ = 0.0064; Fisher’s exact test), and both 17q-Del and LOY cells enriched for HSCs (*P*_adj_ = 2.6 × 10^−14^, *P*_adj_ = 1.0 × 10^−56^; Fisher’s exact test; Fig. 5i and Supplementary Fig. 25).

Having located the mosaic subclones in the scRNA-seq data, we more deeply characterized their molecular phenotypes controlled by cell type. First, gene ontology analysis of the differentially expressed genes between HSCs with and without LOY identifies pathways linked to HSC quiescence^52,53^ (10% FDR; Supplementary Tables 15 and 16), potentially explaining the observed HSC enrichment of LOY in BM712. Next, we confirm a distinct transcriptional profile for 17q-Del cells, with differential activity seen for 16 pathways (Molecular Signatures Database (MSigDB) Hallmark; Supplementary Tables 15 and 16 and Supplementary Fig. 27) including those related to HSPC proliferation, differentiation and metabolism. These pathways include *MYC* and mTOR signaling through *mTORC1*—two known downstream effectors of somatic *NF1* inactivation^49,54^—which can be linked to HSC expansion and inhibition of differentiation^55,56^. Indeed, we find 17q-Del cells are significantly enriched for HSCs compared with 17p-Del cells (*P*_adj_ = 2.1 × 10^−5^; Fig. 5g), potentially mediated through *MYC* and/or *mTORC1* upregulation^55,56^. Finally, 17q-Del cells show an altered DNA damage response, with decreased expression of *BRCA1*, *BRCA2*, *FANCI* and *BLM*—implying these cells might be prone to acquire further alterations. Together, this suggests that BM712 underwent a stepwise acquisition of a potentially ‘higher-risk’ molecular phenotype; first, HSCs were enabled to exit quiescence and bias their differentiation (17p-Del); and, second, cells became more proliferative and HSC-like, and potentially more permissive to acquiring further mutations.

Finally, we explored the presence and functional impact of these mSVs in scRNA-seq data generated from terminally differentiated CD34^−^ blood cells. We annotated 2,965 cells into eight cell types using published reference data^57^ (Fig. 5j and Supplementary Fig. 28), and performed targeted CNA re-calling^58^. Notably, we find a significant enrichment for monocytes in 17p-Del cells (Fig. 5k), a circulating downstream progeny of CMPs. These data underscore that these mSVs, identified in HPSCs, could impact peripheral blood cells. In contrast, our efforts to re-detect CNAs within the smaller 17q-Del region were unsuccessful due to its limited number of expressed genes, underscoring the superior capability of Strand-seq in functionally characterizing mSVs relative to scRNA-seq.

### Functional effects of mSVs in blood samples

**Fig. 6:**
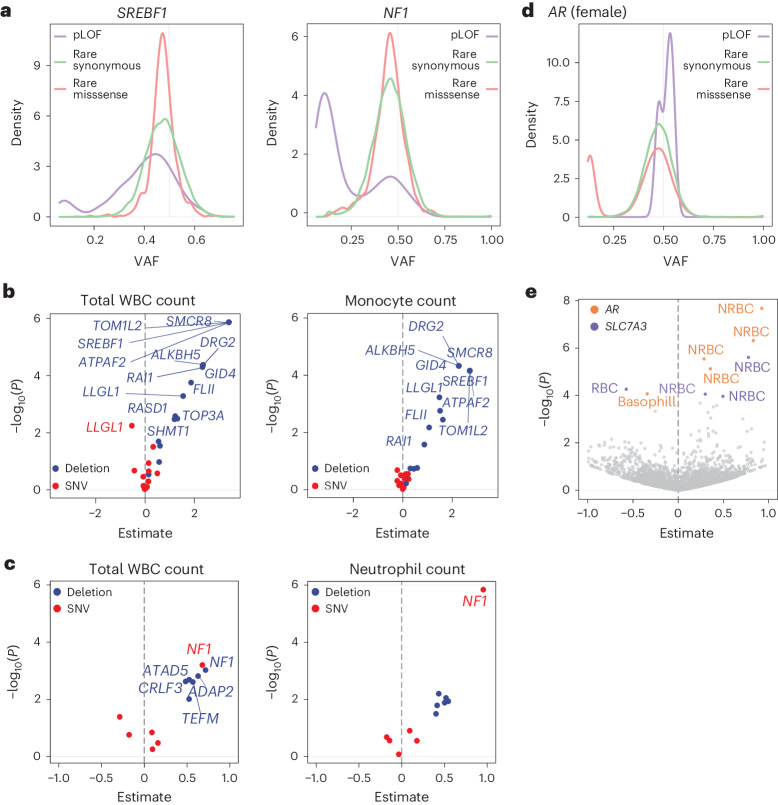
Functional effects of mSVs are supported by re-analysis of UK Biobank data. **a**, VAF plot for SNVs in *SREBF1* and *NF1*, separated by mutation type, in the UK Biobank. **b**,**c**, Volcano plots showing burden test results for genes in the 17p-Del (**b**) and 17q-Del (**c**) (somatic mosaic deletions on chromosomes 17p and 17q) candidate regions, respectively. Genes with *P*_adj_ < 0.05 are labeled. A subset of blood count traits is depicted (see Supplementary Fig. 29 for all blood count traits). **d**, VAF plot for SNVs in *AR*, separated by mutation type, in females (see Supplementary Fig. 34 for males). **e**, Volcano plot showing association test results of single rare missense SNVs at the Xq-Inv (somatic mosaic inversion on chromosome Xq) locus for all 11 blood count traits (generated from female donors). The full respective list of missense variants analyzed is included in Supplementary Table 18. Variants with *P*_adj_ < 0.05 are colored by gene and labeled by trait: NRBC, nucleated red blood cell count; basophil, basophil count; RBC, red blood cell count. Variants with *P*_adj_ ≥ 0.05 are colored in gray. The *y* axes in **b**, **c** and **e** depict nominal *P* values. For **b**, **c** and **e**, *P* values were obtained using the two-sided Wald test followed by the Benjamini–Hochberg multiple correction.

To extrapolate these findings to a larger cohort of blood samples, we interrogated the UK Biobank cohort^59^. The phenotypic data paired with whole-exome sequencing (WES) data from 469,792 donors^59^ provide the opportunity to study somatic mutations in relation to blood counts. Focusing on our top hits—*NF1*, *SREBF1* and *AR*—we extracted rare (minor allele frequency (MAF) < 1%) SNVs and small (<50 bp) insertion and deletion variants (INDELs) from UK Biobank samples, and classified these based on their predicted impact (Supplementary Table 18). Since CNA losses affecting both the 17p-Del and 17q-Del regions were previously documented^2,60^, we additionally made use of WES-based CNA calls^60^ which we analyzed by burden testing (Methods). We first concentrated on the 17p-Del and 17q-Del regions, analyzing gene-disrupting SNVs. We find a bimodal VAF distribution for *NF1* and *SREBF1* predicted loss-of-function (pLoF) SNVs, but not for rare synonymous and rare missense variants (Fig. 6a). These data indicate that gene-disrupting pLoF SNVs represent a common source of mosaicism at these loci. Furthermore, they emphasize the link between gene-disrupting mSVs affecting *SREBF1* and *NF1*, and clonal expansions in normal blood.

Furthermore, at the *SREBF1* locus, we find CNA losses and pLOF SNVs are independently associated with altered blood counts (*n* = 2 losses and *n* = 74 pLOF SNVs; Supplementary Table 18 and Supplementary Figs. 29 and 30), with the *SREBF1* gene being among the strongest hits within the 17p-Del region for several categories, including elevated total leukocytes (*P*_adj_ = 0.00012; loss) and elevated monocytes (*P*_adj_ = 0.0012; loss) (Fig. 6b and Supplementary Fig. 29). These findings independently support that *SREBF1* loss may contribute to a cell-type bias in leukocytes, specifically towards monocytes. When repeating the same analysis for all genes in the 17q-Del region, we find losses at 5 of 6 genes are associated with elevated total leukocytes—yet, only for *NF1* do we observe that both loss and pLoF SNVs are significant (*P*_adj_ = 0.042 for both; Fig. 6c, Supplementary Table 18 and Supplementary Figs. 29 and 30). This supports the contributions of both 17p-Del and 17q-Del to cell-type skewing and potentially clonal expansion in blood. Interestingly, pLOF SNVs in *NF1* are associated with a marked increase in neutrophil counts (*P*_adj_ = 0.00019), strongly implicating this gene in myeloid-skewed hematopoiesis.

Lastly, we analyzed rare missense SNVs at the Xq-Inv locus (*n* = 5 genes), motivated by earlier reports of activating somatic missense mutations in *AR*^61^, which we reasoned could potentially mirror the AR activation molecular phenotype seen in BM65. In females, we observe a bimodal VAF for missense SNVs, but neither for pLoF nor for rare synonymous SNVs, suggesting that *AR* missense SNVs, but not other SNVs, exhibit somatic mosaicism (Fig. 6d and Supplementary Notes). Furthermore, five rare *AR* missense SNVs, but no *AR* pLoF SNVs, are associated with altered blood cell counts (*P*_adj_ < 0.05, for all five SNVs; Fig. 6e). These fall into exon 1 (*n* = 3), exon 2 (*n* = 1) and exon 4 (*n* = 1), all of which also harbor missense SNVs in cancer that impinge on AR function^61^. We observe association with increased nucleated red blood cell count for *n* = 4 missense SNVs (*P*_adj_ < 0.05, for all four), and decreased basophil count for the remaining SNV (*P*_adj_ = 0.043). These findings independently support a link between *AR* activation and altered cell counts in UK Biobank samples.

## Discussion

Our study provides an investigation into the impact of large-scale mosaicisms on normal HPSCs. Using the resolution of Strand-seq (Supplementary Fig. 31), we identify mSVs in most (84%) donors, although mSV subclonal expansion is confined to older (>60) donors. Subclonal mSVs show myeloid cell-type bias and active proliferation pathways, mirroring important features of CH^48^. Therefore, mSVs may represent an important contributor to CH, with their high prevalence potentially accounting for ‘missing’ CH drivers^62^.

Subclonal mSVs are found at diverse loci, yet result in similar dysfunctional signaling pathways, with predominant myeloid-lineage enrichment. This is notable in light of the observation of myeloid skewing in aging HSPCs^28^ and the involvement of myeloid cells in leukemogenesis^63^. Our findings on cell-type biases are bolstered by a recent preprint^64^, which reports an in vivo screen showing pronounced myeloid bias following *NF1* knockout in mouse HPSCs (Supplementary Fig. 32).

The close association of SCEs and mSVs suggests that mSVs frequently arise as a byproduct of DSB repair^21,65^. Intriguingly, mSV formation appears to occur constantly over age, akin to base substitution processes showing consistent activity over life^66^. However, SCE formation slightly reduces with age, perhaps due to altered DNA repair pathway activities^67–69^. Moreover, mosaicism-bearing cells appear more prone to accumulate further mSVs—analogous to CH driven by SNVs where the presence of multiple drivers implies higher cancer susceptibility^70^. Conversely, newly formed singleton mSVs often result in large terminal alterations that do not reach appreciable CF, perhaps due to the detrimental consequences of segmental aneuploidy^13^. Collectively, factors other than increased mSV formation are likely to foster mSV subclonal expansion during aging. The less effective purging of cells comprising mSVs, exhaustion of HSCs decreasing their clonal diversity^12^ or changes in the bone marrow microenvironment may contribute to the subclonal expansion of mSVs in aged donors.

To better understand how mSVs clonally expand in normal blood, additional studies are required. Given its demonstrated ability to discover and functionally characterize mSVs, conducting Strand-seq at scale^71^ could enable future studies in larger cohorts. However, limitations remain: Strand-seq is currently not suited to detecting mSVs <200 kb, and is restricted to dividing cells that can incorporate BrdU^16^. Furthermore, scalable single-cell methods that account for both mSVs and SNVs are lacking, highlighting an area for future technology development.

In conclusion, this study enhances our understanding of how mSVs alter molecular phenotypes in a cell-type-specific manner. Our approach paves new ways for studying mSV landscapes in diverse normal tissues and diseases in the future.

## Methods

### Ethics declarations

For samples from the Department of Hematology and Oncology, Medical Faculty Mannheim, Heidelberg University, the use of primary human materials for research purposes was approved by the Medical Ethics Committee II of the Medical Faculty Mannheim of the Heidelberg University. The Ethics approval number is 2013-509N-MA. For samples from Ulm University Hospital, collection and investigation was approved by the Internal Review Board (Ethikkommission) at Ulm University (392/16). Healthy samples used in this study were obtained from waste bone fragments obtained from endoprosthetic surgery and cardiovascular surgery. Recruitment was based on availability and written, informed consent. The status ‘healthy’ (normal) was defined as being negative for HIV and hepatitis B and C, having a normal blood count and having no history of or currently active malignancy. For samples from the Department of Medicine V, Hematology, Oncology and Rheumatology, University of Heidelberg, bone marrow samples were harvested from the posterior iliac crest. The studies on aging of bone marrow HSPCs have been approved by the Ethics Committee for Human Subjects at the University of Heidelberg. Healthy human subjects were recruited through an announcement published in the Department’s Newsletter for patients and their family. Before donation, healthy subjects were examined and screened by an internist and blood examinations (complete blood count, routine panel of laboratory examinations) were performed to assure their ‘healthy’ status. UCB was collected after informed consent of the mother using the guidelines approved by the Ethics Committee on the use of Human Subjects. All donors provided written, informed consent and all interventions were performed in accordance with the Declaration of Helsinki.

### Human samples

Healthy donor human UCB and bone marrow samples were obtained either as frozen aliquots of mononuclear cells (MNCs) or freshly isolated from Heidelberg University Hospital, Ulm University Hospital, Mannheim University Hospital and ATCC (ATCC PCS-800-013), and were cryopreserved in liquid nitrogen until processing. Strand-seq library generation was initiated from cultures obtained from either freshly isolated or freshly thawed MNCs. For scMNase-seq and scRNA-seq, freshly thawed MNCs were used.

### Statistics and reproducibility

All significance tests used are reported, where applied, in the main text. Multiple testing correction was utilized as required, indicated by *P*_adj_, with an FDR of 10%. No statistical method was used to predetermine sample size. No data were excluded from the analyses. Our targeted analysis of UK Biobank data employed a more stringent significance threshold of *P*_adj_ < 0.05.

### HSPC culturing and Strand-seq library preparation

UCB samples were obtained from Heidelberg University Hospital. Bone marrow was isolated from donor bone marrow aspirations (*n* = 2), discarded pelvis from hip replacement surgeries (*n* = 6) or sternum removed during routine heart surgeries (*n* = 8) (Supplementary Table 1). Cells were stained on ice in the dark for 30 min with CD34-APC (clone 581; BioLegend; 1:100), CD38-PE/Cy7 (clone HB7; eBioscience), CD45Ra-FITC (clone HI100; eBioscience), CD90-PE (clone 5E10; eBioscience) and LIVE/DEAD Fixable Near-IR Dead Cell Stain (ThermoFisher). Single, viable CD34^+^ cells (gating as per Supplementary Fig. 1) were FACS-sorted (BD FACSMelody, 100-μM nozzle, single-cell mode, gates determined using BD FACSDiva 8.0) directly into ice-cold complete medium (Stemspan serum-free expansion medium supplemented with 100 ng ml^−1^ SCF and Flt3 (Stem Cell Technologies) and 20 ng ml^−1^ IL-3, IL-6, G-CSF and TPO (Stem Cell Technologies)). Cells were seeded into Corning Costar Ultra-Low Attachment 96-well plates (Sigma-Aldrich) at a density of 1–2 × 10^5^ cells per ml and cultured for 42 h in the presence of BrdU (40 μM). BrdU-containing nuclei were sorted into 96-well plates and subjected to Strand-seq using the standard library preparation protocol^16^, which includes treatment with MNase for DNA fragmentation. Strand-seq libraries were generated using a Biomek FXP liquid handling robotic system^16,22^, and sequenced on an Illumina NextSeq 500 sequencing platform (MID-mode, 75-base pair (bp) paired-end sequencing).

### scMNase-seq

HSPCs from a healthy bone marrow donor were obtained from ATCC (ATCC PCS-800-013), and UCB samples as described above. Frozen MNCs were thawed and stained as per Supplementary Table 5, with antibodies outlined in Supplementary Table 19, to distinguish the eight distinct HSPC populations outlined in Supplementary Fig. 6a. Single, viable HSPCs (gating strategy Supplementary Fig. 6b) were index-sorted using a BD FACSAria Fusion Cell Sorter (100-μM nozzle, single-cell mode) into 96-well plates containing 5 μl of modified freeze buffer (0.1% NP-40, 7.5% dimethylsulfoxide, 42.5% 2X Profreeze-CDM (Lonza) in PBS) and frozen. ScMNase-seq^78^ libraries were generated from sorted, frozen single cells as per Strand-seq library preparation^22^, with the following modification: the Hoechst/ultraviolet treatment step was omitted (with scMNase-seq requiring no BrdU incorporation). Following single-cell sequencing, each cell had an average coverage of 613,483 uniquely mapped fragments.

### Building nucleosome occupancy reference set cell-type classifiers

The scNOVA framework enables cell-typing of each Strand-seq library, which is achieved by subjecting nucleosome occupancy patterns produced through MNase digestion to machine learning-based classification^20^. While previously applied to distinguish cell lines from distinct tissues^20^, here we employed this approach to classify closely related HSPC cell types, based on generating single-cell nucleosome occupancy reference profiles from scMNase-seq data. To achieve this, we index-sorted both the bone marrow- and UCB-derived CD34^+^ cells from eight HSPC cell types using previously defined immunophenotypes^24^ (Supplementary Fig. 6a and Supplementary Table 5), as described above. Indexed scMNase-seq libraries were used as the ground-truth input for cell-type classifiers. In the case of bone marrow HSPCs, the gene-body nucleosome occupancy profiles were extracted for 305 high-quality single cells and normalized by library size to obtain reads per million. These normalized values were log_2_-transformed and standardized, before being subjected to supervised PLS-DA^79^ to (1) identify informative feature sets, and subsequently (2) build a classification model. To identify informative feature (gene) sets for each cell type, we used variable autosomal genes to build an *X*-matrix (305 cells × 18,851 genes) and a *Y*-matrix (305 cells × 8 cell types). These *X* and *Y* variables were passed to the PLS-DA feature selection process, which outputs variance importance in projection (VIP) scores for each feature. In total, 1,904 genes with a VIP score >90% of the null distribution from the permutation test were retained for the second stage of feature selection. In the second feature selection stage, an additional *X*-matrix (305 cells × 1,904 genes) and *Y*-matrix (305 cells × 1 cell type; with cell type in this case being binary information for each cell either belonging to that cell type (1) or not (0), based on FACS indexes) were passed to the PLS-DA, and features with a VIP score >95% of the null distribution from the permutation test retained. This was repeated for each cell type, resulting in a final informative feature set of 819 marker genes (Supplementary Table 7b). We repeated these steps for 175 high-quality single cells obtained from UCB HSPCs, which resulted in 899 marker genes as significant feature sets for cell-type classification (Supplementary Table 7a). We constructed distinct nucleosome occupancy-based classifiers for bone marrow and UCB HSPCs based on nucleosome occupancy patterns in the gene bodies of selected marker genes for cells derived from each source (Supplementary Table 7a,b and Code availability).

### mSV discovery in Strand-seq data

We utilized the scTRIP computational approach^14^ for single-cell mSV discovery, to identify duplications, deletions, inversions, whole chromosome aneuploidies and complex mSVs. This approach leverages the synergy of three distinct readouts—read depth, strand and haplotype phase—retrieved from Strand-seq data, for haplotype-aware mSV discovery. We performed segmentation of the Strand-seq data by jointly processing strand-resolved binned read depth data across all single cells of a sample, used as a multivariate input signal with a squared-error assumption^14^. The single-cell footprints of different mSV classes (derived from unique combinations of read depth, strand and phase) were then discovered using scTRIP (achieved by running the ‘MosaiCatcher’ pipeline with default settings)^14^. This approach uses a Bayesian framework to compute posterior probabilities for each mSV diagnostic footprint, and to derive haplotype-resolved mSV genotype likelihoods. Each diagnostic footprint translates into the expected number of copies sequenced in Watson (W) and Crick (C) orientation, contributing to a respective genomic segment. The framework distinguishes between WC and CW chromosomal ground states, and is thus haplotype-aware. It implicitly allows us to perform mSV discovery throughout the genome, including for chromosomes sequenced only on the C strand (CC ground state) or such sequenced only on the W strand (WW ground state), since unambiguous single-cell mSV footprints exist for each ground state^14^. The framework estimates clonal frequency levels for each mSV and uses them to define prior probabilities for each candidate mSV. In this way, the framework benefits from the observation of mSVs in more than one cell, enabling improved detection of mSVs in subclones^14^—in addition to facilitating the detection of singleton mSVs. In contrast to CNAs, balanced inversions and translocations must be present in at least two single cells to trigger an mSV call^14^. We verified that the frequency of singleton mSVs detected using Strand-seq is consistent with results from intermediate coverage single-cell WGS (Supplementary Fig. 33). This suggests that short-term cell culturing with BrdU does not introduce singleton mSVs.

### Cell-type enrichment testing

We devised cell-type enrichment tests for each of the identified subclones exhibiting specific mSVs, using a control group consisting of all individuals over the age of 60 who were not affected by mSVs. We performed a binomial test to determine if the number of cells in a particular cell type within the subclone was greater than expected, based on the cell-type composition of the control group. We then calculated permutation-based adjusted *P* values for each subclonal mSV by randomly sampling the same number of HSPCs from the entire single-cell population 100,000 times and tallying the number of cells from given cell types in question belonging to that subclone.

### Single-cell multiomic analysis of differential gene activities in HSPC subclones

Differentially active genes in subclones affected by mSVs were identified in the Strand-seq data using scNOVA^20^. We used scNOVA’s infer altered gene activity module with the PLS-DA option, which is recommended for the investigation of low-CF subclones^20^. To regress-out cell-type effects in the identification of differential gene activity, we considered predicted cell type for each single cell as a confounding factor when we executed the infer altered gene activity module. Genes within the respective deleted region were masked, to avoid spurious associations^80^. Genes with significantly altered gene activity (10% FDR) were subjected to gene set over-representation analysis using ConsensusPathDB^77^. Using this approach, certain pathways may exhibit a significant *P* value for both upregulated and downregulated genes, with some genes contained in ConsensusPathDB functioning as activators and others as suppressors. Over-represented pathways (FDR 10%) were visualized as dot plots. When comparing 17p-cells and wild-type (WT) cells in BM712 in Fig. 5f, we considered all cells carrying the 17p-Del, including those harboring other mosaicisms in addition to 17p-Del, as ‘17p-cells’.

### Investigation of potential *cis*-effects of a balanced inversion

To investigate the local effects of Xq-Inv in BM65, we employed scNOVA^20^. We utilized a sliding window approach suitable to uncover the *cis-*effects of balanced mSVs, resolved by haplotype^20^. We focused on the Xq-Inv-affected segment, including both of its rearranged TADs. We first defined CREs based on a previous study utilizing the assay for transposase-accessible chromatin with sequencing (ATAC-seq) in HSPCs^24^. We used a sliding window (300 kb in size, moving 10 kb each)^20^, analyzing CREs along chromosome X, to infer chromosome-wide haplotype-specific nucleosome occupancy for the mSV subclone and WT cells, which is predictive for chromatin accessibility^20^. For each sliding window, haplotype-specific nucleosome occupancy values at CREs from the mSV subclone (nucleosome occupancy in the active X chromosome/nucleosome occupancy in the inactive X) and WT cells (nucleosome occupancy in the active X/nucleosome occupancy in the inactive X) were compared using likelihood ratio tests to obtain nominal *P* values *[P real]*. As a multiple testing correction to control the type I error, we performed a permutation test by randomly shuffling genotype labels of each single cell (mSV or WT) in the single-cell reads per million matrix 1,000 times. For each permutation, we performed likelihood ratio tests to compare nucleosome occupancy between randomly shuffled mSV subclones and WT cells. We computed the number of incidences we observed with the same, or a lower, *P* value than *[P real]* from 1,000 permutations, and divided this value by the number of trials (*n* = 1,000) to estimate the permutation-adjusted *P* value. Sliding windows with permutation-adjusted *P* value lower than 0.1 were identified as significantly altered windows, and were assigned to the nearest genes within the same TAD boundaries.

### scRNA-seq

Bone marrow MNCs were thawed and stained as described above, with the following antibodies: CD34-AF488 (clone 561; BioLegend; 1:20), CD38-PE/Cy7 (clone HB7; eBioscience; 1:100). Cells were washed and resuspended as above, and stained for 5 min with DAPI before sorting. The gating strategy as described in Supplementary Fig. 1 was used to sort CD34^+^ cells and CD34^−^ cells, respectively, into ice-cold 0.04% BSA in PBS using a BD FACSMelody cell sorter. For each donor, two samples were prepared: one sample of CD34^+^ cells and one sample a 50:50 mixture of CD34^+^ and CD34^−^ cells. scRNA-seq libraries for each sample were generated as per the standard 10X Genomics Chromium 3′ (v.3.1 chemistry) protocol. Completed libraries were sequenced on a NextSeq5000 sequencer (HIGH mode, 75-bp paired-ends).

### scRNA-seq data processing, unsupervised clustering and cell-type annotation

Transcripts were aligned to GRCh38 and quantified into count matrices using Cellranger mkfastq and count workflows (10X Genomics, v.3.1.0, default parameters). Seurat^81^ (v.3.2.2) was used for quality control of single cells and unbiased clustering of the data. Briefly, cells with <1,000 unique molecular identifiers (UMIs) and cells with >6% of mitochondrial reads were removed as ‘low quality’. Normalization, feature selection, scaling and dimensionality reduction were carried out using default settings. To annotate cell types, previously reported scRNA-seq data from HSPCs^51^ were used as a reference for cell-type labeling using SingleR^72^. Differential expression analysis to identify cluster-/genotype-specific marker genes was carried out using the FindMarkers() function from Seurat.

### Targeted CNA re-calling in scRNA-seq data

scRNA-seq data were normalized to counts per million (CPM) and transformed into log_2_(CPM/10 + 1) using Seurat^81^ (v.3.2.2). These values were then subject to targeted CNA re-calling using the CONICSmat package^58^, as described previously^20^. For the analysis of donor BM712, all three subclonal mosaicisms were investigated: 17p-Del, 17q-Del and LOY. By default, the CONICSmat ‘plotChrEnrichment’ function considers regions with more than 100 expressed genes for CNA discovery. Since we performed targeted re-calling of CNAs previously identified with the high-breakpoint mapping resolution of Strand-seq, we considered regions with five or more expressed genes in our analysis. The numbers of expressed genes detected per mSV were as follows: 17p-Del: 24 genes; 17q-Del: 5 genes; LOY: 17 genes for CD34^+^ dataset; 17p-Del: 28 genes; 17q-Del: 5 genes; LOY: 38 genes for CD34^−^ dataset (Supplementary Table 14). To profile CNA regions, CONICSmat generates distributions of average expression levels across single cells in the given regions, and then fits one-component and two-component mixture models to these distributions. It further compares the likelihood ratios of being one-component (unimodal; that is, absence of CNAs) and two-component (bimodal; that is, presence of CNAs), to determine the most-likely state in those regions based on the Bayesian information criterion. Candidate CNA regions identified as likely to be bimodal within a 1% FDR criterion (based on a Chi-squared likelihood ratio test) were considered further for downstream analysis. Once the region was inferred to have bimodality, the posterior probability for each single cell to belong to the normal clone or CNA subclone was calculated. A posterior probability cutoff of 0.8 was used to assign single cells into one of the two clones. This analysis was repeated for each subclonal mosaicism event.

### SCE mapping and locus-specific SCE enrichment

We constructed genome-wide maps of SCEs in each single cell by subjecting the Strand-seq data of single cells to the MosaiCatcher pipeline^14^, followed by manual inspection and curation of each call yielding SCE positional coordinates for each cell. Candidate SCEs were identified as changes in strand-state (for example, WW to WC) on a chromosome, whereby we conservatively focused on chromosomes showing only a single changepoint. Chromosomes bearing singleton mSVs were removed by manual inspection, unless the observed strand-state patterns were clearly not attributed to an mSV alone (for example, a terminal deletion together with a complete change in strand orientation, such as WW to C), signifying the co-occurrence of an mSV and an SCE. Coordinates were padded by 1 bp upstream and downstream. GRCh38 was divided into 500-kb bins using the bedtools makewindows command^82^, and overlaps between these 500-kb bins and our SCE callset were generated using bedtools intersect, giving the number of times each bin is hit by an SCE. A bin was considered to be hit if the majority of an SCE confidence interval fell within that bin, and each SCE was only counted in a single bin. To compute significance of the calculated SCE counts per bin, the count data per bin genome-wide were then fit to a negative binomial distribution using the fitdist function from fitdistrplus^83^, and *P* values calculated using the qnbinom function (with size = 1.2506716, mu = 0.4823156), applying Benjamini–Hochberg correction. To compute overlap of mSV breakpoints with SCEs, we considered 200-kb-sized breakpoint regions (reported breakpoints ±100 kb).

### Breakpoint refinement by WGS

Bulk genomic DNA was isolated from CD34^−^ cells (viable cells from the donors that were not put into culture to be used for Strand-seq library preparation) using the QIAamp DNA Blood Maxi Kit as per the manufacturer’s instructions. Samples were sequenced using a NextSeq5000 (HIGH mode, 75-bp paired-end). Raw WGS reads were aligned to GRCh38 using bwa (v.0.7.15), sorted, marked for duplicates and indexed. mSVs were called using Delly2 (default parameters), combining split read, paired-end and read depth analysis^76^. Unfiltered mSV calls were compared with our callset. Since split read analysis failed to identify the precise breakpoints of the 17p-Del that reside in a repeat-rich region, we generated a single, directional composite bam file of this region based on our Strand-seq data to allow for 17p-Del breakpoint refinement with BreakpointR^33^.

### UK Biobank analysis

#### Data collection

The UK Biobank is a population database of approximately half a million participants^59^. For SNVs and INDELs, we used the population-level exome OQFE variants for 469,792 individuals (UK Biobank field ID 23157). For autosomal large deletions, we used CNA loss calls on WES data that were recently generated by subjecting 200,624 individuals from the UK Biobank to the CNest copy-number caller^60^. We considered CNA calls >1 kb. Additionally, we obtained phenotypic data for 11 blood count traits (UK Biobank category ID 100081), containing count for white blood cells, basophils, eosinophils, monocytes, neutrophils, lymphocytes, red blood cells, nucleated red blood cells, platelets, reticulocytes and high-light-scatter reticulocytes. This research was conducted under the application number 83497. The UK Biobank has ethics approval from the North West Multi-centre Research Ethics Committee (21/NW/0157).

#### Variant annotation

We annotated SNVs/INDELs from WES data using Variant Effect Predictor (VEP v.1.0.3) with the Loss-Of-Function Transcript Effect Estimator (LOFTEE v.0.3-beta) plugin. Variant annotation was performed using Hail v.0.2. According to annotation results, we grouped variants into rare loss of function variants (‘high confidence’ identified by LOFTEE with a MAF < 1%) and rare missense variants (missense variants annotated by VEP with MAF < 1% in the UK Biobank cohort). In the case of CNA losses, we considered deletions overlapping coding exons with MAF < 1%.

#### Association testing

The blood count data were rank normalized using the ‘RNOmni’ package in R^84^. Linear regression models (blood count ~ genotype + covariates) were used to assess the association between three loci of interest (17p-Del, 17q-Del and Xq-Inv) and blood counts adjusted for several covariates, including age, sex and the first five principal components derived from genotype arrays. For all genes at the respective 17p and 17q loci, we used gene rare pLoF burden and rare large CNA loss burden as genotype in the regression model. For all genes at the X chromosomal locus of interest, we used gene burden for rare pLoF variants and rare missense variants in the model. Moreover, since missense variants can have distinct functional impacts, we also performed single-variant association analysis for rare missense mutations at the Xq-Inv locus by sex. The volcano plot in Fig. 6e presents nominal *P* values derived solely from female donors, since we made the observation of sex-biased VAF distributions at the *AR* locus in UK Biobank samples. For all data, see Supplementary Fig. 34. A minimum of three individuals with relevant variants was required for association tests of a given gene, with the exception of the 17p-Del CNA seen in only two UK Biobank donors based on WES. *P* values were obtained using the Wald test and the Benjamini and Hochberg method was used to correct for multiple hypothesis testing.

### Reporting summary

Further information on research design is available in the Nature Portfolio Reporting Summary linked to this article.

## Online content

Any methods, additional references, Nature Portfolio reporting summaries, source data, extended data, supplementary information, acknowledgements, peer review information; details of author contributions and competing interests; and statements of data and code availability are available at 10.1038/s41588-024-01754-2.

### Supplementary information

Supplementary Information Supplementary Figs. 1–37, Methods, Notes and references.

Reporting Summary

Supplementary Data Strand-seq plots of singleton mSVs.

Supplementary Table All supplementary tables associated with this study.

## Data Availability

All genomics data generated in this study (Strand-seq, scMNase-seq, scRNA-seq, bulk WGS) are available under the following accession: EGAS00001006567. We re-analyzed publicly available bulk RNA-seq and bulk ATAC-seq data from HSPCs (GSE75384) to characterize signature genes while building the scMNase-seq-based cell-type classifier, and to define CREs in the HSPCs. Additionally, we utilized publicly available databases as follows: Molecular Signatures Database (MSigDB; https://www.gsea-msigdb.org/gsea/msigdb/), ConsensusPathDB (http://cpdb.molgen.mpg.de/).

## Code availability

Our study has made publicly available scMNase/nucleosome occupancy-based classifiers for cell-typing Strand-seq libraries in HSPCs. This MATLAB-based classifier can be accessed and downloaded from GitHub, to facilitate its use in research studies (https://github.com/jeongdo801/NO_based_HSPC_classifier). The MATLAB code can be converted to other platforms (R/python) using openly accessible tools (such as large language models). To facilitate open access, the list of signature genes and their weights have also been made available in an open text format through the same hyperlink.
