# Supplementary material for: Cell-type-specific consequences of mosaic structural variants in hematopoietic stem and progenitor cells
Source: Nat Genet. 2024 May 28;56(6):1134–46. doi: 10.1038/s41588-024-01754-2 (PMC11176070; doi:10.1038/s41588-024-01754-2)
Supplement: Supplementary file 2 — Reporting Summary [file 41588_2024_1754_MOESM2_ESM.pdf]

nature portfolio

Corresponding author(s): Ashley D. Sanders and Jan O. Korbel

Last updated by author(s): Mar 17, 2024

Reporting Summary

Nature Portfolio wishes to improve the reproducibility of the work that we publish. This form provides structure for consistency and transparency in reporting. For further information on Nature Portfolio policies, see our Editorial Policies and the Editorial Policy Checklist.

Statistics

For all statistical analyses, confirm that the following items are present in the figure legend, table legend, main text, or Methods section.

n/a	Confirmed	
☐	☒	The exact sample size (*n*) for each experimental group/condition, given as a discrete number and unit of measurement
☒	☐	A statement on whether measurements were taken from distinct samples or whether the same sample was measured repeatedly
☐	☒	The statistical test(s) used AND whether they are one- or two-sided<br>*Only common tests should be described solely by name; describe more complex techniques in the Methods section.*
☐	☒	A description of all covariates tested
☐	☒	A description of any assumptions or corrections, such as tests of normality and adjustment for multiple comparisons
☐	☒	A full description of the statistical parameters including central tendency (e.g. means) or other basic estimates (e.g. regression coefficient) AND variation (e.g. standard deviation) or associated estimates of uncertainty (e.g. confidence intervals)
☐	☒	For null hypothesis testing, the test statistic (e.g. *F*, *t*, *r*) with confidence intervals, effect sizes, degrees of freedom and *P* value noted<br>*Give P values as exact values whenever suitable.*
☒	☐	For Bayesian analysis, information on the choice of priors and Markov chain Monte Carlo settings
☒	☐	For hierarchical and complex designs, identification of the appropriate level for tests and full reporting of outcomes
☒	☐	Estimates of effect sizes (e.g. Cohen's *d*, Pearson's *r*), indicating how they were calculated

Our web collection on statistics for biologists contains articles on many of the points above.

Software and code

Policy information about availability of computer code

Data collection	No data collection was performed
Data analysis	The computational code of our cell type classifiers is hosted on GitHub (see https://github.com/jeongdo801/Bonemarrow_HSPC_classifier). All code is available freely for academic research.<br>Other software used:<br>Mosaicatcher (https://github.com/friendsofstrandseq/mosaicatcher-pipeline), scNOVA (https://github.com/jeongdo801/scNOVA), StrandPhaseR (https://github.com/daewoooo/StrandPhaseR), CONICSmat (https://github.com/diazlab/CONICS), NucTools (https://homeveg.github.io/nuctools), Delly2 (https://github.com/dellytools/delly), STRING (https://string-db.org/), BWA (v0.7.15), STAR (v2.7.9a), SAMtools (v1.3.1), biobambam2 (v2.0.76), deeptools (v2.5.1), perl (v5.16.3), Python (v3.7.4), cuDNN (v7.6.4.38), CUDA (v10.1.243), TensorFlow (v1.15.0), scikit-learn (v0.21.3), matplotlib (v3.1.1), R (v4.1.1), FlowJo, BD FACSDiva, fitdistrplus (v1.1.6), regioneR (v1.24.0), monocle3(v1.3.1), Seurat (v3.2.2), MultiK (v0.1.0), gamlss (v5.4.1), SingleR (v4.3), escape (v3.18), BD FACSDiva (v8.0), Rnomi (https://github.com/zrmacc/RNOmni), STRING (https://string-db.org/)

For manuscripts utilizing custom algorithms or software that are central to the research but not yet described in published literature, software must be made available to editors and reviewers. We strongly encourage code deposition in a community repository (e.g. GitHub). See the Nature Portfolio guidelines for submitting code & software for further information.

Data

Policy information about availability of data

All manuscripts must include a data availability statement. This statement should provide the following information, where applicable:

- Accession codes, unique identifiers, or web links for publicly available datasets
- A description of any restrictions on data availability
- For clinical datasets or third party data, please ensure that the statement adheres to our policy

All genomics data generated in this study (Strand-seq, scMNase-seq, scRNA-seq, bulk WGS) are available under the following accession: EGAS00001006567.
We re-analysed publicly available bulk RNA-seq and bulk ATAC-seq data from HSPCs (GSE75384) to characterise signature genes while building scMNase-seq based cell-type classifier, and to define cis-regulatory elements (CRE) in the HSPCs.
Additionally, we utilised publicly available database as follows: Molecular signature database (MSigDB; https://www.gsea-msigdb.org/gsea/msigdb/), ConsensusPathDB (http://cpdb.molgen.mpg.de/).

Research involving human participants, their data, or biological material

Policy information about studies with human participants or human data. See also policy information about sex, gender (identity/presentation), and sexual orientation and race, ethnicity and racism.

Reporting on sex and gender	In this study, male and female sexes of donors were defined based on 2 layers on information: 1) the sex reported from the clinician who collected the samples and 2) based on the sex chromosome content of the majority of cells from a given donor. Donors in which all cells had a monosomy of the X chromosome, and at least 25% of cells containing a Y chromosome, were considered male. Donors which had a disomy of the X chromosome in the majority of cells, and no cells with a Y chromosome, were considered female.
Reporting on race, ethnicity, or other socially relevant groupings	No such grouping have been made in this study.
Population characteristics	No population-based characteristics have been used in this study
Recruitment	Healthy human subjects were recruited either through an announcement published in the Department's Newsletter for patients and their family and/or through availability and informed consent.<br>We did not bias the selection of donor samples, yet, enriched our cohort for donors older than 60 given prior data showing an abundance of subclonal CNAs in the blood of donors from that age range.
Ethics oversight	For samples from the department of Hematology and Oncology, Medical Faculty Mannheim, Heidelberg University, the use of primary human materials for research purposes was approved by the Medical Ethics Committee II of the Medical Faculty Mannheim of the Heidelberg University. The Ethics approval number is 2013-509N-MA. For samples from Ulm University Hospital, collection and investigation was approved by the Internal Review Board (Ethikkomission) at Ulm University (392/16). Healthy samples used in this study were obtained from waste bone fragments obtained from endoprosthetic surgery and cardiovascular surgery. Recruitment was based on availability and written informed consent. The status "healthy" was defined as being negative for HIV, Hepatitis B and C, having a normal blood count and no history or currently active malignancy. For samples from the Department of Medicine V, Hematology, Oncology and Rheumatology, University of Heidelberg, bone marrow samples were harvested from the posterior iliac crest. The studies on aging of bone marrow HSPCs have been approved by the Ethics Committee for Human Subjects at the University Heidelberg. Before donation, healthy subjects were examined and screened by an internist and blood examinations (complete blood count, routine panel of laboratory examinations) were performed to assure their "healthy" status. UCB was collected after informed consent of the mother using the guidelines approved by the Ethics Committee on the use of Human Subjects.<br><br>All donors provided written informed consent and all interventions were performed in accordance with the Declaration of Helsinki.

Note that full information on the approval of the study protocol must also be provided in the manuscript.

Field-specific reporting

Please select the one below that is the best fit for your research. If you are not sure, read the appropriate sections before making your selection.

☒ Life sciences ☐ Behavioural & social sciences ☐ Ecological, evolutionary & environmental sciences

For a reference copy of the document with all sections, see nature.com/documents/nr-reporting-summary-flat.pdf

Life sciences study design

All studies must disclose on these points even when the disclosure is negative.

Sample size	No sample-size calculation was performed, since this study focuses on initial detection and functional characterisation of a class of mutations,

Sample size	rather than on performing statistical tests between groups of samples. The cohort size was determined by the number of healthy BM/UCB samples available
Data exclusions	We excluded low quality single-cell libraries that showed very low (<200,000 unique reads), uneven coverage, or an excess of 'background reads' yielding noisy Strand-seq data prior to analysis. scMNase-seq cells were excluded based on extremely high or low coverage, indicative of multiple cells or low quality. Finally, scRNA-seq cells were excluded based on having either < 1000 UMIs or > 6 % of reads mapping to the mitochondrial genome
Replication	Since these experiments involved limited samples from healthy donors, experiments were not replicated or repeated. However, it is reasonable to assume that findings would be reproducible in cohorts of similar donors.
Randomization	Does not apply, as there are no experimental groups defined in our study
Blinding	Does not apply, as this study focuses on intra-sample comparison rather than performing statistical tests between groups of samples

Reporting for specific materials, systems and methods

We require information from authors about some types of materials, experimental systems and methods used in many studies. Here, indicate whether each material, system or method listed is relevant to your study. If you are not sure if a list item applies to your research, read the appropriate section before selecting a response.

Materials & experimental systems

n/a	Involved in the study	
☐	☒	Antibodies
☒	☐	Eukaryotic cell lines
☒	☐	Palaeontology and archaeology
☒	☐	Animals and other organisms
☒	☐	Clinical data
☒	☐	Dual use research of concern
☒	☐	Plants

Methods

n/a	Involved in the study	
☒	☐	ChIP-seq
☐	☒	Flow cytometry
☒	☐	MRI-based neuroimaging

Antibodies

Antibodies used	ACS (clone, manufacturer, catalogue number, lot number): APC mouse anti-human CD34 (clone 581; Biolegend; #343509; Lot: B260867), PeCy7 mouse anti-human CD38 (clone HB7; eBioscience; #15538396; Lot: 1974952), FITC mouse anti-human CD45Ra (clone HI100; eBioscience; #15526406; Lot: 4329359), PE mouse anti-human CD90 (clone 5E10; eBioscience; # 15526836; Lot:1982684), PE-Cy5 mouse anti-human CD2 (clone RPA-2.10; BD Biosciences; #555328; Lot: 7123718), PE-Cy5 mouse anti-human CD3 (clone HIT3a; BD Biosciences; #561007; lot: 8163944), PE-Cy5 mouse anti-human CD4 (clone RPA-T4; BD Biosciences; #15840679; lot: 9016960), PE-Cy5 mouse anti-human CD7 (clone M-T701; BD Biosciences; #555362; lot: 7058673), PE-Cy5 mouse anti-human CD8 (RPA-T8; BD Biosciences; #15861499; lot: 7179955), APC-Cy7 mouse anti-human CD10 (clone HI10a; Biolegend; #312212; lot: B242546), PE-Cy5 mouse anti-human CD11b (clone ICRF44; BD Biosciences; #555389; lot: 8171911), PE-Cy5 mouse anti-human CD14 (clone 61D3; eBioscience; #15014942; lot: 4330408), PE-Cy5 mouse anti-human CD16 (clone 3G8; BD Biosciences; #555408; lot: 8261948), PE-Cy5 mouse anti-human CD19 (clone HIB19; BD Biosciences; #555414; lot: 8183956), PE-Cy5 mouse anti-human CD20 (clone 2H7; BD Biosciences; #561761; lot: 8324650), PE-Cy5 mouse anti-human CD56 (clone B159; BD Biosciences; #561904; lot: 7177552), BV605 mouse anti-human CD123 (clone 7G3; BD Biosciences; #564197; lot: 8092987), PE-Cy5 mouse anti-human GPA (clone GA-R2; BD Biosciences; #559944; lot: 7199932)
Validation	All antibodies were validated for the specific application by the manufacturer and validation data is available on the manufacturer's website.<br>FACS<br>CD34 CD34 https://www.biolegend.com/fr-ch/products/apc-anti-human-cd34-antibody-6090 DOI: 10.1538/expanim.49.97<br>CD38 CD38 https://www.thermofisher.com/antibody/product/CD38-Antibody-clone-HB7-Monoclonal/25-0388-42 DOI: 10.1016/j.stem.2021.02.001<br>CD45Ra PTPRC https://www.thermofisher.com/antibody/product/CD45RA-Antibody-clone-HI100-Monoclonal/14-0458-82 DOI: 10.1080/2162402X.2017.1371399<br>CD90 THY1 https://www.thermofisher.com/antibody/product/CD90-Thy-1-Antibody-clone-eBio5E10-5E10-Monoclonal/12-0909-42<br>CD2 CD2 https://www.bdbiosciences.com/en-de/products/reagents/flow-cytometry-reagents/research-reagents/single-color-antibodies-ruo/pe-cy-5-mouse-anti-human-cd2.555328 PMCID: PMC1384357<br>CD3 CD3 https://www.bdbiosciences.com/en-de/products/reagents/flow-cytometry-reagents/research-reagents/single-color-antibodies-ruo/pe-cy-5-mouse-anti-human-cd3.561007 DOI: 10.1002/eji.1830110412<br>CD4 CD4 https://www.fishersci.fr/shop/products/anti-cd4-pe-cy-5-clone-rpa-t4-bd/15840679/en PMCID: PMC1384357<br>CD7 CD7 https://www.bdbiosciences.com/en-de/products/reagents/flow-cytometry-reagents/research-reagents/single-color-antibodies-ruo/PE-Cy%25252525E2%2525252584%25252525A25-Mouse-Anti-Human-CD7.555362 PMID: 7506726<br>CD8 CD8 https://www.bdbiosciences.com/en-de/products/reagents/flow-cytometry-reagents/research-reagents/single-color-antibodies-ruo/pe-cy-5-mouse-anti-human-cd8.555368 doi: 10.1084/jem.190.11.1627<br>CD10 MME https://www.biolegend.com/en-us/products/apc-cyanine7-anti-human-cd10-antibody-4034?GroupID=BLG5905 doi.org/10.1084/jem.181.6.2271<br>CD11b CD11b https://www.bdbiosciences.com/en-de/products/reagents/flow-cytometry-reagents/research-reagents/single-color-

antibodies-ruo/pe-cy-5-mouse-anti-human-cd11b.555389 PMID: 2416682
CD14 CD14 https://www.thermofisher.com/antibody/product/CD14-Antibody-clone-61D3-Monoclonal/15-0149-42 DOI: 10.1128/IAI.00381-07
CD16 CD16 https://www.bdbiosciences.com/en-de/products/reagents/flow-cytometry-reagents/research-reagents/single-color-antibodies-ruo/pe-cy-5-mouse-anti-human-cd16.555408 https://doi.org/10.1073/pnas.79.10.3275
CD19 CD19 https://www.bdbiosciences.com/en-de/products/reagents/flow-cytometry-reagents/research-reagents/single-color-antibodies-ruo/pe-cy-5-mouse-anti-human-cd19.555414 https://doi.org/10.4049/jimmunol.151.6.2915
CD20 CD20 https://www.bdbiosciences.com/en-de/products/reagents/flow-cytometry-reagents/research-reagents/single-color-antibodies-ruo/pe-cy-5-mouse-anti-human-cd20.555624 https://doi.org/10.1002/cyto.990140212
CD56 NCAM-1 https://www.bdbiosciences.com/en-de/products/reagents/flow-cytometry-reagents/research-reagents/single-color-antibodies-ruo/pe-cy-5-mouse-anti-human-cd56-ncam-1.561904 https://doi.org/10.1084/jem.184.5.1845
CD123 CD123 https://www.bdbiosciences.com/en-de/products/reagents/flow-cytometry-reagents/research-reagents/single-color-antibodies-ruo/bv605-mouse-anti-human-cd123.564197 https://doi.org/10.1073/pnas.90.23.11137
GPA CD235a https://www.bdbiosciences.com/en-de/products/reagents/flow-cytometry-reagents/research-reagents/single-color-antibodies-ruo/pe-cy-5-mouse-anti-human-cd235a.559944 https://doi.org/10.3109/10428199409049629

Plants

Seed stocks

Report on the source of all seed stocks or other plant material used. If applicable, state the seed stock centre and catalogue number. If plant specimens were collected from the field, describe the collection location, date and sampling procedures.

Novel plant genotypes

Describe the methods by which all novel plant genotypes were produced. This includes those generated by transgenic approaches, gene editing, chemical/radiation-based mutagenesis and hybridization. For transgenic lines, describe the transformation method, the number of independent lines analyzed and the generation upon which experiments were performed. For gene-edited lines, describe the editor used, the endogenous sequence targeted for editing, the targeting guide RNA sequence (if applicable) and how the editor was applied.

Authentication

Describe any authentication procedures for each seed stock used or novel genotype generated. Describe any experiments used to assess the effect of a mutation and, where applicable, how potential secondary effects (e.g. second site T-DNA insertions, mosiacism, off-target gene editing) were examined.

Flow Cytometry

Plots

Confirm that:

☒ The axis labels state the marker and fluorochrome used (e.g. CD4-FITC).

☒ The axis scales are clearly visible. Include numbers along axes only for bottom left plot of group (a 'group' is an analysis of identical markers).

☒ All plots are contour plots with outliers or pseudocolor plots.

☒ A numerical value for number of cells or percentage (with statistics) is provided.

Methodology

Sample preparation

Bone marrow mononuclear cells were isolated either from the sternum or hip during either heart surgery, hip replacement or bone marrow aspiration and frozen until processing. Umbilical cord blood was obtained from the umbilicus of normal births, and frozen until processing. All samples were then processed as follows: cryopreserved cells were thawed rapidly at 37 C and resuspended dropwise in 10 ml warm Roswell Park Memorial Institute (RPMI) medium with 100 µg/ml Dnase I. Cells were centrifuged for 5 mins at 300 g, and resuspended in ice-cold phosphate buffered saline (PBS) with 2% foetal bovine serum (FBS) and 5mM EDTA. Samples were then stained on ice in the dark for 30 mins as follows: for Strand-seq, cells were s stained with CD34-APC (clone 581; Biolegend), CD38-PeCy7 (clone HB7; eBioscience), CD45Ra-FITC (clone HI100; eBioscience), CD90-PE (clone 5E10; eBioscience), and LIVE/DEAD™ Fixable Near-IR Dead Cell Stain (Thermofisher). For scMNase-seq, cells were stained with a lineage cocktail (CD2-PE-Cy5, RPA-2.10, BD Biosciences; CD3-PE-Cy5, HIT3a, BD Biosciences; CD4-PE-Cy5, RPA-T4, BD Biosciences; CD7-PE-Cy5, M-T701, BD Biosciences; CD8-PE-Cy5, RPA-T8, BD Biosciences; CD11b-PE-Cy5, ICRF44, BD Biosciences; CD14-PE-Cy5, 61D3, eBioscience; CD16-PE-Cy5, 3G8, BD Biosciences; CD19-PE-Cy5, HIB19, BD Biosciences; CD20-PE-Cy5, 2H7, BD Biosciences; CD56-PE-Cy5, B159, BD Biosciences; GPA-PE-Cy5, GA-R2, BD Biosciences), CD10-APC-Cy7 (clone HI10a; Biolegend), CD123-BV605 (clone 7G3; BD Biosciences) CD34-APC (clone 581; Biolegend), CD38-PeCy7 (clone HB7; eBioscience), CD45Ra-FITC (clone HI100; eBioscience), CD90-PE (clone 5E10; eBioscience), and LIVE/DEAD™ Fixable Near-IR Dead Cell Stain (Thermofisher). After staining, cells were washed once in 4 ml ice-cold PBS with 2% FBS and 5 mM EDTA and centrifuged at 300 g for 5 mins. Cells were resuspended in ice-cold PBS with 2% FBS and 5 mM EDTA for sorting.

Instrument

BD FACSAria™ Fusion Cell Sorter, BD FACSMelody™

Software

FlowJo, BD FACSDiva™

Cell population abundance

Due to limited sample material, post-sort purities were not re-assessed using flow cytometry.

Gating strategy

For Strand-seq: The first gate excluded any cellular debris based on FSC-A vs SSC-A. These cells were then sub-gated to identify only Single Cells, based on removal of outliers from the SCC-W vs SSC-A plot. Viable Cells were gated within the Single Cells based on a low intracellular staining for the viability stain Fixable LIVE/DEAD near-IR (Fixable LIVE/DEAD near-IR Viability

vs FSC-A). Finally, the ultimate sorting population of CD34+ (and CD34-) cells was gated based on a high (or low) expression of CD34 (CD34-APC vs CD38-PeCy7). The full gating strategy is depicted in Supplemental Figure S1.
For scMNase-seq: The first gate excluded any cellular debris based on FSC-A vs SSC-A (. Viable Cells were gated within the based on a low intracellular staining for the viability stain Fixable LIVE/DEAD near-IR (Fixable LIVE/DEAD near-IR Viability vs FSC-A). Lineage-negative cells were isolated from the viable cells by gating for cells with the lowest expression of a custom lineage panel of antibodies (CD2, CD3, CD4, CD7, CD8, CD10, CD11b, CD14, CD16, CD19, CD20, CD56, GPA; Lineage-PeCy5). CD34+CD38+ cells were gated based on a high expression of CD34 and CD38; whereas CD34+CD38- were gated based on a high expression of CD34 and low expression of CD38 (CD34-APC vs CD38-PeCy7). Within the CD34+CD38+ population, the final gate for CLPs was defined based on a high expression of CD10 (CD45Ra-FITC vsCD10-APCCy7). CD10- cells were further gated into final populations of MEPs (CD45Ra-CD123-), CMPs (CD45Ra-CD123mid), GMPs (CD45Ra+CD123mid) and pDCs (CD45Ra+CD123hi) (CD45Ra-FITC vs CD123-BV605). Within the CD34+CD38- population, final gates were defined for HSCs (CD45Ra-CD90+), MPPs (CD45Ra-CD90-), and LMPPs (CD45Ra+CD90-) (CD45Ra-FITC vs CD90-PE). The full gating strategy is depicted in Supplemental Figure S8.

☒ Tick this box to confirm that a figure exemplifying the gating strategy is provided in the Supplementary Information.
